# Small molecule-assisted assembly of multifunctional ceria nanozymes for synergistic treatment of atherosclerosis

**DOI:** 10.1038/s41467-022-34248-y

**Published:** 2022-11-01

**Authors:** Xiaoxue Fu, Xiaojuan Yu, Junhao Jiang, Jiaxin Yang, Lu Chen, Zhangyou Yang, Chao Yu

**Affiliations:** Chongqing Key Laboratory for Pharmaceutical Metabolism Research, Chongqing Pharmacodynamic Evaluation Engineering Technology Research Center, College of Pharmacy, Chongqing Medical University, 400016 Chongqing, P. R. China

**Keywords:** Vascular diseases, Nanoparticles, Drug delivery

## Abstract

Considering that intravascular reactive oxygen species (ROS) and inflammation are two characteristic features of the atherosclerotic microenvironment, developing an appropriate strategy to treat atherosclerosis by synergistically regulating ROS and inflammation has attracted widespread attention. Herein, a special molecule, zoledronic acid, containing imidazole and bisphosphonate groups, was selected for the first time to assist the assembly of cerium ions and produce functionalized ceria-zoledronic acid nanocomposites (CZ NCs). It not only serves as a new carrier for different kinds of drugs (e.g. probucol, PB) but also exerts an efficient multienzyme activity to achieve collaborative therapy. More importantly, platelet membrane-coated biomimetic nanoplatform (PCZ@PB NCs) specifically accumulate at inflammatory atherosclerotic lesions, synergistically regulate ROS levels and inflammation, and efficiently inhibit foam cell formation. This novel assembly method can also be applied in the treatment of many other diseases associated with oxidative stress and inflammation.

## Abstract (web summary)

Regulating reactive oxygen species and inflammation are important for treating atherosclerosis. Here, the authors create a cell membrane coated zoledronic acid ceria nanocomposite loaded with probucol for synergistic drug and scavenging nanozyme treatment of atherosclerosis demonstrating application in vivo.

## Introduction

Atherosclerosis (AS) is characterized by increased intravascular reactive oxygen species (ROS) production, lipid peroxidation and inflammation throughout the disease procession and is a leading cause of vascular diseases worldwide^1,2^. The regulation of oxidative stress and expression levels of the inflammatory molecules in the AS microenvironment is particularly important^3,4^. Various anti-inflammatory agents have been assessed for treating AS^5–7^. However, their nonspecific distribution, rapid clearance and low efficacy in ROS removal have limited their therapeutic efficacy in AS treatment^8,9^. Thus, there is an urgent need to develop alternative strategies for targeted drug delivery and effective treatment of AS.

Nanomaterial-based drug delivery strategies provide a promising platform for AS treatment as they enhance drug efficacy and reduce toxic side effects^10,11^. However, most nanomaterials lack a multifunctional collaborative strategy and are designed to deliver drugs into plaques using their passive targeting characteristics^12,13^. Numerous multifunctional nanoplatforms have been recently fabricated, which not only serve as drug carriers but also as therapeutic agents to synergistically treat AS, further increasing their therapeutic efficacy^14,15^. Despite the potential advantages of the abovementioned design, several problems persist in its practical application, such as the relatively complex construction of nanocarriers, unsatisfactory therapeutic effects, need for long-term drug administration, and lack of an active targeting design^16–18^. These limitations warrant the development of a novel nanotherapeutic strategy to achieve synergistic treatment of AS.

Owing to the unique and numerous enzyme activities (e.g., superoxide dismutase [SOD], catalase [CAT], phosphatase, oxidase, etc.) of ceria nanozymes, the development of an assembly technology to construct functional ceria nanozyme platforms for treating AS is a promising strategy^19,20^. In fact, ceria nanozymes demonstrate numerous important applications in treating oxidative stress and inflammatory diseases such as Alzheimer’s disease, ischemic stroke, and rheumatoid arthritis^21–24^. However, their poor biodegradability and lack of drug-carrying capacity limit application in treating certain diseases, such as AS. Although the ceria metal organic framework (Ce-MOF) is endowed with a drug loading ability, its enzyme activity is significantly reduced because of its large (>200 nm) and irregular size^25–29^. This limited enzyme activity can be attributed to certain physicochemical properties of cerium ions and the lack of functional linker molecules or technological strategies^30,31^. To the best of our knowledge, there has been no study reporting a ceria nanozyme and their drug delivery system-based treatment of AS. Hence, studies aiming to explore the construction of functional linking molecules and develop an assembly technology are important for maintaining the multifunctional enzyme activity and drug delivery capacity of ceria nanozymes. Such studies will further expand to the treatment of oxidative stress and inflammatory diseases, particularly of AS.

**Fig. 1:**
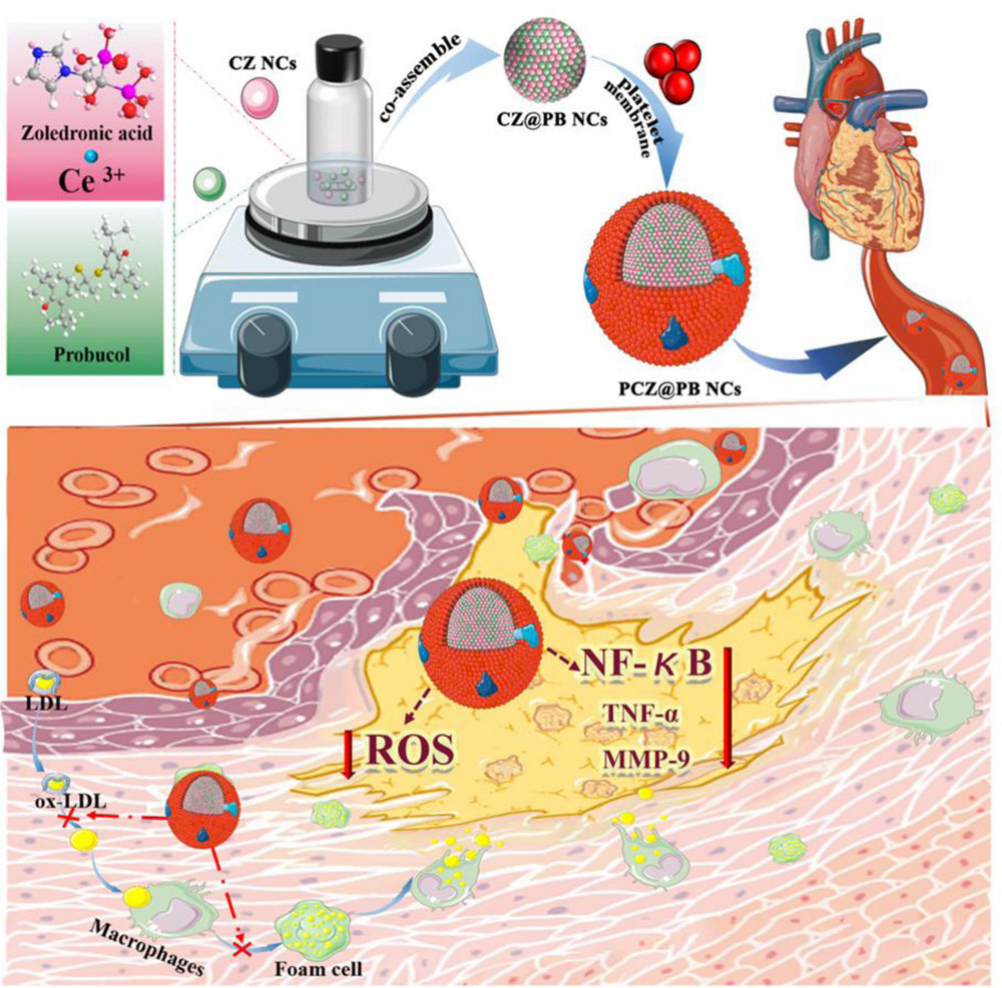
The preparation and application of the nanozyme (platelet membrane-coated biomimetic nanoplatform, PCZ@PB NCs). Biomimetic probucol-loaded nanozyme is obtained by assembly of zoledronic acid, cerium ions and probucol and encapsulated in the platelet membranes. After administration, the PCZ@PB NCs were enriched to plaques through passive accumulation as well as active targeting of inflammatory lesions, reducing foam cell formation, resisting inflammation and removing excess ROS.

Here, a multifunctional ceria nanozyme assembly is constructed with the assistance of a small molecule and enabled efficient AS treatment. Zoledronic acid (ZOL), which comprises imidazole and a bisphosphonate group, was used for the first time to promote the assembly of cerium ions. The functionalized ceria-ZOL nanocomposites (CZ NCs) exhibit the advantages of traditional ceria nanozymes and Ce-MOFs. These functional nanozymes reportedly serve as a drug loading and delivery platform, reduce drug toxicity, and allow multienzyme activity with drugs to ameliorate the oxidative stress and inflammatory microenvironment of AS. Importantly, the biomimetic modification of the platelet membrane undoubtedly enhances the biosafety of nanodrugs while endowing them with active and passive dual targeting functions (Fig. 1). This work might provide new perspectives in designing precision medicine for treating of oxidative stress and inflammatory diseases.

## Results

### Design, preparation, and characterization of ceria nanozymes

Ceria nanozymes exhibit excellent properties for biological applications (Supplementary Fig. 1). Although Ce-MOF demonstrates a drug-carrying ability owing to its pore size^32^, it does not effectively maintain nanozyme activity because of its large (>200 nm) and irregular size. Initially, alendronate, which comprises a bisphosphonate group, was used to chelate cerium ions^33^, whereas the functional molecule dimethylimidazole (HMIM) was selected to construct a dual-ligand ceria nanozyme platform (CHA). The atomic force microscopy (AFM) characterization of the platforms generated under different conditions of synthesis (Supplementary Figs. 2–3, Supplementary Table 1) showed that CHA was obtained via the novel coassembly of cerium ions, alendronate acid (AL) and HMIM (Supplementary Video 1). The experiments characterizing the material implied that CHA exhibited a uniform size of ~10 nm (Supplementary Fig. 4). The drug loading experiments showed that although CHA effectively loaded some dyes (Supplementary Fig. 5) through the involvement of multiligand molecules, its ability to load many other drugs was poor (as observed via the transmission electron microscopy [TEM] of probucol [PB], Supplementary Fig. 6), limiting its further biomedical applications.

**Fig. 2:**
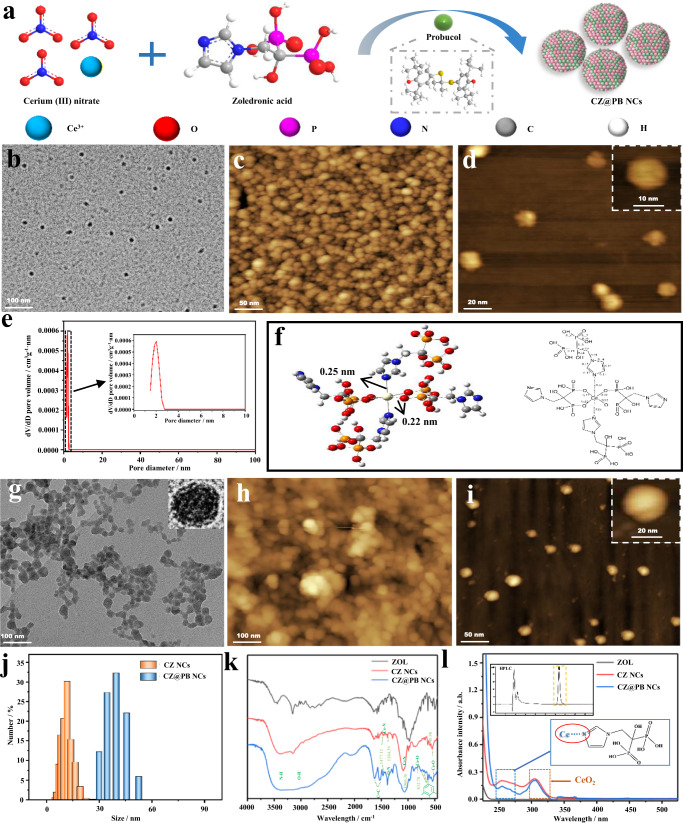
Preparation and characterization of CZ NCs and CZ@PB NCs. **a** Schematic illustrating the preparation of CZ@PB NCs. **b** TEM image of CZ NCs, and **c**, **d** AFM images of CZ NCs. **e** Pore size distribution of CZ NCs. **f** Molecular simulation of CZ NCs. **g** TEM image of CZ@PB NCs, and **h**, **i** AFM images of CZ@PB NCs. **j** Size distribution. **k** Infrared spectra. **l** UV–vis absorption spectra. CZ NCs ceria-zoledronic acid nanocomposites, CZ@PB NCs CZ NCs assembled with probucol, TEM transmission electron microscopy, AFM atomic force microscopy. All the experiments were repeated for three times (*n* = 3) with similar results.

The smart molecule ZOL, which combines the high affinities of imidazole and bisphosphonate groups with different metal ions, can be used to mediate the assembly of metal nanozymes. As shown in Fig. 2a and Supplementary Video 2, ZOL not only self-assembled with cerium ions to form CZ NCs but also coassembled with Zn^2+^, Mn^2+^, Fe^2+^, and Cu^2+^ to form multimetal nanocomposites. The range of their hydrated particle sizes was from 3 nm to 1.5 μm according to the dynamic light scattering (DLS) measurements (Supplementary Fig. 7). Optimizing synthesis conditions showed that gradually increasing cerium ion concentration (the molar ratio of ZOL:Ce is <4, cerium ion concentration >2.5 mM) leads to a large amount of precipitation due to the lack of protection of the system by ZOL, which is not conducive to the synthesis and stability of ceria nanozymes. Further DLS analyses demonstrated that the generation of CZ NCs from cerium ions at a concentration of 2.5 mM produces a homogeneous and stable particle size. Subsequent experiments determined that the concentration of cerium nitrate was 2.5 mM (Supplementary Fig. 8). The morphology of CZ NCs was characterized (Fig. 2b–d, j and Supplementary Fig. 9), and the results showed that CZ NCs were dispersed as homogeneous spheres with an average size of ~10 nm, and DLS showed a hydration particle size of ~15 nm (PDI 0.164). X-ray diffraction results showed that CZ NCs exist primarily in the amorphous state. Nitrogen adsorption experiments confirmed the presence of microporous structures with a pore size of ~2 nm in CZ NCs (Fig. 2e and Supplementary Fig. 10). The results of the subsequent thermogravimetric analysis of CZ NCs are shown in Supplementary Fig. 11. In contrast to the imidazole aromatic ring, which exhibits weak chemical bonding, and hence, almost completely decomposes at low temperatures (120–190 °C)^28^, CZ NCs showed a lower rate of pyrolysis, with ~60% of the residue remaining at 800 °C. The interaction between cerium and ZOL and the resulting structural network in the prepared CZ NCs enhances the thermal stability.

To further explore the self-assembly mechanism of ZOL with cerium ions, molecular simulations were performed (Fig. 2f). Density functional theory calculations were performed using Gaussian 09 package of programs. 6-31G(d) basis set was used for C, H, O, N, and P. Stuttgart RSC ANO basis set was employed to incorporate relativistic effects on the cerium metal atom. The simulations suggest that the MOF-like structure was formed mainly by cerium ions with ZOL, and four ZOL molecules were attached around each cerium ion to form an orderly framework structure. The coordinate bond lengths of each cerium ion to the imidazole group and the phosphonic acid group were calculated to be 0.25 and 0.22 nm, respectively. The actual drug loading capacity of CZ NCs was evaluated based on the MOF-like structure. Various dyes or drugs were coassembled with CZ NCs and characterized using UV‒vis spectroscopy or DLS (Supplementary Fig. 12 and Supplementary Table 2). The particle size of ceria nanodrugs increased with the molecular weight and hydrophobicity of the coreacting molecules, indicating that CZ NCs can potentially serve as a versatile platform for coassembly with numerous other drugs; however, the exact mechanism of this coassembly warrants further investigation.

A typical antioxidant drug molecule, probucol (PB), was chosen for coassembly with CZ NCs to obtain CZ@PB NCs. The TEM and AFM images of CZ@PB NCs demonstrated a clear spherical structure, whose size increased from 10 nm (CZ NCs) to 25 nm (Fig. 2g–i), and the hydrodynamic size also increased from 15 to 40 nm (Fig. 2j, PDI 0.208). The infrared (IR) spectroscopy results showed that both CZ NCs and CZ@PB NCs presented typical IR absorption peaks for cerium ion-imidazole groups and Ce-O (Fig. 2k). Additionally, CZ@PB NCs possess a characteristic benzene ring absorption peak due to the addition of PB (Fig. 2k), and the UV‒Vis spectra showed characteristic absorption peaks for Ce-N and Ce-O, corroborating the IR spectra (Fig. 2l). The characteristic absorption peak of PB obtained using high-performance liquid chromatography (HPLC) confirmed the presence of PB (Fig. 2l, insert). A standard curve of PB was first plotted using HPLC to further quantify its concentration in CZ@PB NCs (Supplementary Fig. 13); the encapsulation rate of PB was 39%, and its release curve was plotted (Supplementary Fig. 14).

### Construction and characterization of PCZ@PB NCs with multienzyme activities

**Fig. 3:**
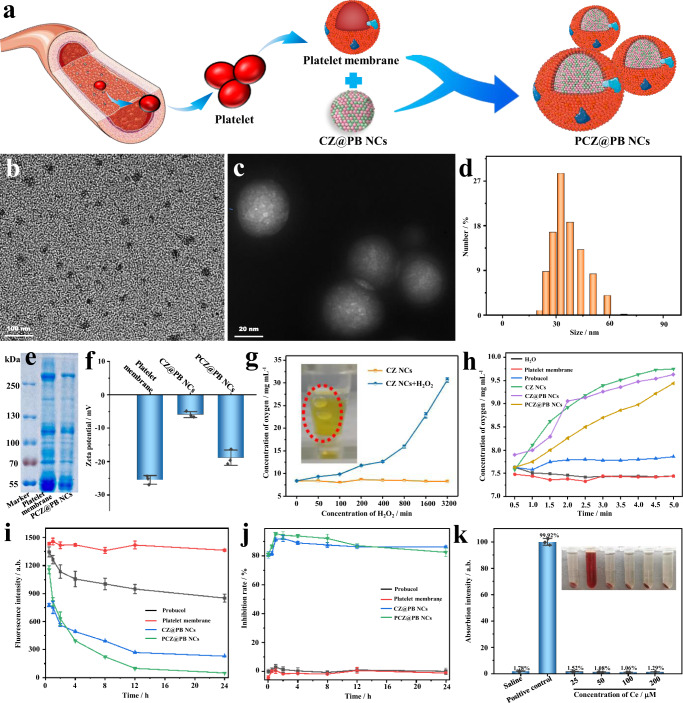
Characterization of the biomimetic nanodrugs PCZ@PB NCs, and verification of their multienzyme activity. **a** Schematic illustrating the preparation of PCZ@PB NCs with a platelet membrane coating on CZ@PB NCs. **b** TEM image of PCZ@PB NCs. **c** TEM image of PCZ@PB NCs negatively stained with uranyl acetate. **d** Size distribution. **e** Representative protein bands stained with Coomassie brilliant blue. **f** Zeta potential. **g** Characterization of the ability of CZ NCs to catalyze oxygen production from different concentrations of hydrogen peroxide (H_2_O_2_). **h** Determination of different components catalyzing H_2_O_2_ (1 mM) into oxygen within 5 min. **i** Staining with the fluorescent probe 2’,7’-dichlorofluorescein-diacetate (DCFH-DA) to characterize the concentration of H_2_O_2_ in the solution over 24 h. **j** Sodium dismutase (SOD) activity measurement. **k** Characterization of the blood compatibility of PCZ@PB NCs. CZ NCs ceria-zoledronic acid nanocomposites, CZ@PB NCs CZ NCs assembled with probucol, TEM transmission electron microscopy. All the experiments were repeated for three times (*n* = 3) and data were presented as mean ± s.d. Analysis of mean zeta potential, dissolved oxygen concentration, mean DCFH fluorescence, superoxide anion inhibition rate and hemolysis rate were performed using one-way ANOVA. *P ≤ 0.05, **P ≤ 0.01, and ***P ≤ 0.001. Specific *P*-values are shown in the source data. Source data are provided as source data files.

The construction of biomimetic nanoplatforms is crucial for investigating the active targeted delivery and biocompatibility of drugs. In this study, platelet membranes were extracted and used to encapsulate CZ@PB NCs, thereby generating biomimetic PCZ@PB NCs (Fig. 3a). TEM and negative staining demonstrated that the platelet membrane encapsulation increased the dispersion of CZ@PB NCs compared with that before coating (Fig. 3b, c). Interestingly, the hydrodynamic size of PCZ@PB NCs was reduced by ~10% (35 nm, PDI 0.213) following encapsulation, potentially due to the compression of CZ@PB NCs by the negative charge of the membrane (Fig. 3d). The protein bands of PCZ@PB NCs stained with Coomassie brilliant blue matched the representative protein bands of the platelet membranes (Fig. 3e). Moreover, Fig. 3f depics that the surface charge of the PCZ@PB NCs (−18.86 mV) obtained after membrane encapsulation was considerably more negative than that of CZ@PB NCs (−5.91 mV) because of the strong negative electrical properties of the platelet membranes. The larger absolute value of the zeta potential was associated with a more stable system, implying that encapsulation increased the stability of CZ@PB NCs. The results of the Western blot analysis showed no significant difference in the expression of the platelet-characteristic GPVI protein and activated characteristic protein before and after platelet encapsulation (Supplementary Fig. 15). Therefore, PCZ@PB NCs will not cause platelet activation leading to thrombi. No significant change in the particle size was observed, implying that the PCZ@PB NCs were stable in H_2_O. However, the particle size significantly increased at pH 5 because of the disintegration typical of MOF-like materials (Supplementary Fig. 16a). PCZ@PB NCs exhibited good stability in both phosphate-buffered saline (PBS) and PBS+fetal bovine serum systems, with no significant change in particle size over 24 h. Moreover, the stability of CZ@PB NCs was substantially affected by the lack of platelet membrane modification, and their hydrated particle size increased by 86% and 93% in the PBS and PBS+fetal bovine serum systems, respectively (Supplementary Fig. 16b). Overall, the biomimetic modification of the platelet membrane undoubtedly enhanced the biostability of nanodrugs while endowing them with multifunctional synergy.

The surface valence distribution of ceria nanozymes determines the type and strength of their enzyme-mimicking activities. Therefore, the valence distributions of CZ NCs and PCZ@PB NCs were analyzed using X-ray photoelectron spectroscopy, and the results showed that the Ce^3+^/Ce^4+^ distributions on the surface of the nanozymes were 51.7%/48.3% and 44.8%/55.2%, respectively, suggesting that both exhibited the potential to stimulate CAT and SOD activities (Supplementary Fig. 17, Supplementary Tables 3–4). CZ NCs were first selected to catalyze the production of hydrogen peroxide (H_2_O_2_) production. The concentration of dissolved oxygen catalyzed by CZ NCs increases gradually with increasing H_2_O_2_ concentration in the system (Fig. 3g). Concurrently, clear bubbles and a color change of the system from colorless to yellow were observed (Fig. 3g insert). Furthermore, we evaluated the changes in the levels of dissolved oxygen in different systems mixed with H_2_O_2_ for 5 min, and CZ NCs, CZ@PB NCs, and PCZ@PB NCs all significantly catalyzed the generation of oxygen from H_2_O_2_ (Fig. 3h). Additionally, fluorescent probes were used to indicate the amount of H_2_O_2_ remaining in the system and visualize its consumption. The fluorescence spectrophotometry results showed that the fluorescence intensity of the CZ NCs, CZ@PB NCs and PCZ@PB NCs groups gradually decreased over time, indicating that the H_2_O_2_ in the system was gradually consumed (Fig. 3i).

Because the superoxide anion (•O_2_^-^), which is a type of ROS, is overproduced in inflammatory diseases, it is necessary to examine whether ceria nanoplatforms exhibit SOD mimetic activity to eliminate •O_2_^-^. The inhibition of •O_2_^-^ production by the nanozymes was characterized using the azole blue method. Based on the results presented in Fig. 3j, the inhibition of •O_2_^-^ production by CZ@PB NCs and PCZ@PB NCs reached more than 80% within 24 h, indicating their excellent SOD enzyme-mimetic activity. Hence, CZ NCs, CZ@PB NCs and PCZ@PB NCs successfully inherited the CAT and SOD enzyme-mimetic activities of the ceria nanoplatform, indicating their good application potential. Notably, the hemocompatibility assay showed that PCZ@PB NCs maintained reassured blood safety even when the cerium ion concentration reached 200 μM (Fig. 3k). These results revealed successful encapsulation by platelet membranes and good hemocompatibility of PCZ@PB NCs, thereby laying the foundation for their future applications.

### In vitro cytotoxicity studies

Macrophages, endothelial cells and smooth muscle cells are associated with atherogenesis, and the safety of CZ NCs, CZ@PB NCs and PCZ@PB NCs in these cells was subsequently evaluated (Supplementary Fig. 18). The CCK-8 analysis showed that the viability of RAW 264.7 cells was greater than 95% after 24 h of incubation with different concentrations of CZ NCs. Conversely, high CZ@PB NCs concentrations decreased cell viability. Furthermore, platelet membranes allowed the cells to maintain a high viability following PCZ@PB NCs treatment. Moreover, the viability of human umbilical vein endothelial cells (HUVECs) was no less than 75% even at cerium ion concentrations of up to 400 μM following 24 h of incubation with CZ NCs and CZ@PB NCs. The potential explanations for these results are that, platelet encapsulation effectively shielded the cells from direct contact with the high concentration of CZ@PB NCs, thereby, reducing any toxicity. Furthemore, the provascular cell proliferation effect exhibited by cerium ions was consistent with that reported in the literature^34,35^. In vascular endothelial cells (VSMCs), neither CZ@PB NCs nor PCZ@PB NCs exhibited substantial toxicity. Overall, PCZ@PB NCs were not significantly cytotoxic, even at relatively high doses, and the platelet membrane coating enhanced cell safety.

### In vitro cellular uptake of PCZ@PB NCs by macrophages

**Fig. 4:**
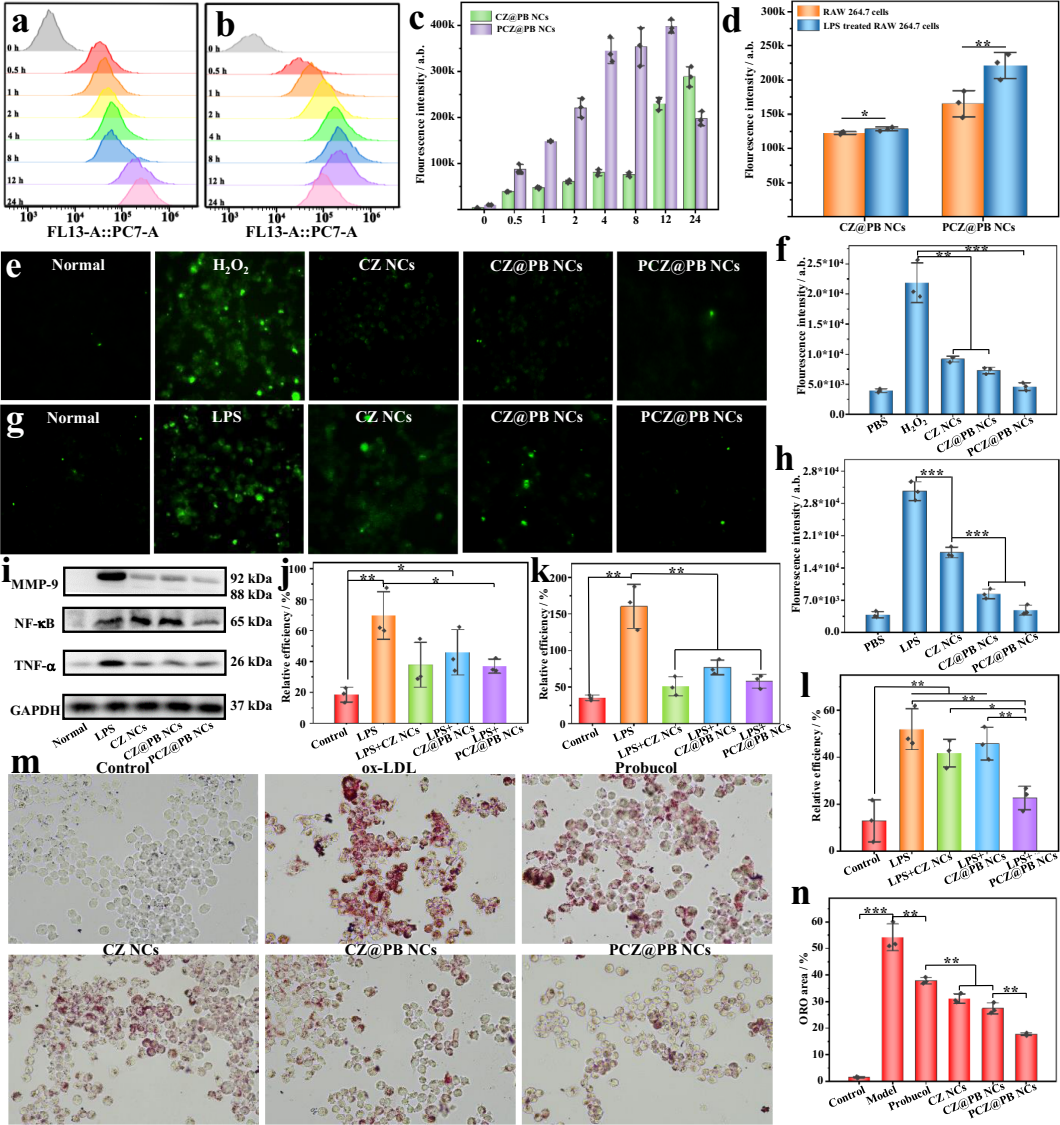
In vitro effects of the biomimetic nanodrug PCZ@PB NCs. **a** Uptake of CZ@PB NCs and **b** PCZ@PB NCs by RAW 264.7 cells within 24 h. **c** Quantification of uptake based on fluorescence intensity. **d** Uptake of CZ@PB NCs and PCZ@PB NCs in RAW 264.7 cells and inflammatory RAW 264.7 cells. Intracellular hydrigen peroxide (H_2_O_2_) levels were characterized using the DCFH-DA probe. **e** Cellular fluorescence imaging. **f** Fluorescence intensity quantified using flow cytometry. Intracellular reactive oxygen species generation following treatment with different formulations and stimulation with Lipopolysaccharide (LPS). **g** Cellular fluorescence imaging. **h** Graph of the fluorescence intensity quantified using flow cytometry. **i**–**l** Western blot analysis of the intracellular expression levels of proinflammatory factors. **j** Tumor necrosis factor-alpha (TNF-α) expression level, **k** Matrix metalloproteinase-9 (MMP-9) expression level, **l** Nuclear factor kappa-B (NF-κB) expression level. **m** Oil red O (ORO) staining to characterize lipid droplets in foam cells. **n** Quantification of foam cells. Scale bars, 20 μm. DCFH-DA 2′,7′-dichlorofluorescein-diacetate, CZ@PB NCs ceria-zoledronic acid nanocomposites assembled with probucol, PCZ@PB NCs platelet membrane coating on CZ@PB NCs. All the experiments were repeated for three times (*n* = 3) and data were presented as mean ± s.d. Analysis of mean fluorescence intensity, relative grayscale, and ORO staining area was performed using one-way ANOVA. *P ≤ 0.05, **P ≤ 0.01, and ***P ≤ 0.001. Specific *P*-values are shown in the source data. Source data are provided as source data files.

Macrophages are vital in the atherosclerotic process, and the formation of foamy macrophages increases inflammation. Therefore, the ability of PCZ@PB NCs to enter macrophages and function effectively inside them is crucial for their utilization in AS treatments. We first investigated the intracellular transport pathway of PCZ@PB NCs in RAW 264.7 cells using confocal microscopy. After 8 h of treatment, we observed the colocalization of fluorescent signals from IR 780 (red) and LysoTracker (green), indicating that endocytosed PCZ@PB NCs were transported primarily via the lysosomal pathway (Supplementary Fig. 19). Flow cytometry was used to study the uptake of PCZ@PB NCs by RAW 264.7 cells at different timepoints. RAW 264.7 cells treated with IR 780-labeled CZ@PB NCs and PCZ@PB NCs demonstrated a gradual increase in the mean fluorescence intensity with an increase in incubation time over 12 h, while the cells showed greater uptake of the membrane-coated PCZ@PB NCs over 24 h. (Fig. 4a–c). Additionally, since macrophages in atherosclerotic plaques are in an inflammatory microenvironment in the presence of numerous proinflammatory mediators, we also explored the uptake of PCZ@PB NCs by normal and lipopolysaccharide (LPS)-prestimulated RAW 264.7 cells. Flow cytometry quantitatively confirmed the internalization profiles of CZ@PB NCs and PCZ@PB NCs in macrophages. Excitingly, the uptake of PCZ@PB NCs in the RAW 264.7 cells was significantly greater than that of CZ@PB NCs, attributable to the high affinity of platelet membranes for inflammatory cells (Fig. 4d). Hence, PCZ@PB NCs are effectively taken up and are more easily internalized by RAW 264.7 cells in the inflammatory state than CZ@PB NCs.

### ROS-scavenging and anti-inflammatory activities of PCZ@PB NCs in macrophages

Excess ROS triggers oxidative stress, causing cell and tissue damage and subsequent inflammation. Therefore, the ROS-scavenging effect of PCZ@PB NCs was investigated. The fluorescent dye DCFH-DA was used to determine the ROS levels; PBS- and H_2_O_2_-treated cells were used as a normal control and ROS overproduction model groups. The H_2_O_2_-treated cells contained higher levels of ROS (green fluorescence), whereas the PCZ@PB NCs-treated group showed the weakest fluorescence intensity following treatment with different formulations (Fig. 4e, f). Moreover, the fluorescence signal of the macrophages was significantly diminished following treatment with different doses of PCZ@PB NCs, similar to that of normal controls, indicating that even low concentrations of PCZ@PB NCs exhibited a strong CAT-mimicking ability and scavenged almost all the applied H_2_O_2_ (Supplementary Figures 20–22). In another model group, RAW 264.7 cells were pretreated with LPS, and the fluorescence intensity of the model group indicated higher ROS levels. Similarly, the PCZ@PB NCs treatment group showed the weakest fluorescence intensity after treatment with different agents (Fig. 4g, h). Hence, the resuls of fluorescence microscopy and flow cytometry analyses show that PCZ@PB NCs dose-dependently inhibits ROS production in RAW 264.7 cells (Supplementary Figs. 23–25).

### In vitro anti-inflammatory activity of PCZ@PB NCs

Because AS is a typical chronic inflammatory disease, alleviating inflammation is crucial for its treatment. Although the ROS-scavenging ability of PCZ@PB NCs was detected, further analysis of their ability to regulate inflammation in RAW 264.7 cells is needed. The expression levels of inflammatory factors in macrophages were analyzed using Western blotting (Fig. 4i–l). LPS-stimulated macrophages from the model group showed a significant increase in the secretion of proinflammatory cytokines. Additionally, CZ@PB NCs and PCZ@PB NCs were added to the cells and coincubated with LPS, and then the proteins were extracted to characterize the expression levels of the typical inflammatory factors and cellular proteins involved in related pathways in the cells. The intracellular nuclear factor-κB (NF-κB) P65 protein phosphorylation level was significantly reduced. The expression levels of the proinflammatory factors, including tumor necrosis factor-alpha (TNF-α) and matrix metalloproteinase-9 (MMP-9), were downregulated in the PCZ@PB NCs-treated group than in the LPS-treated group. This result implies that PCZ@PB NCs attenuate inflammation in RAW 264.7 cells by inhibiting the NF-κB pathway.

### Effects of PCZ@PB NCs treatment on foam cell formation

The macrophage foam cell formation is one of the main hallmarks of atherosclerotic lesions; therefore, we stained the intracellular lipid droplets with oil red O (ORO) and examined whether PCZ@PB NCs treatment effectively inhibited foam cell formation. The RAW 264.7 macrophages treated with oxidized low-density lipoprotein (oxLDL) showed a considerable number of intracellular lipid droplets and significantly more macrophage foam cell formation (Fig. 4m, n). Moreover, macrophage foam cells were attenuated following treatment with PB and CZ NCs. However, foam cell formation was significantly inhibited via treatment with nanocomposites and drugs, particularly PCZ@PB NCs.

### In vivo plaque targeting and tissue distribution of nanodrugs

**Fig. 5:**
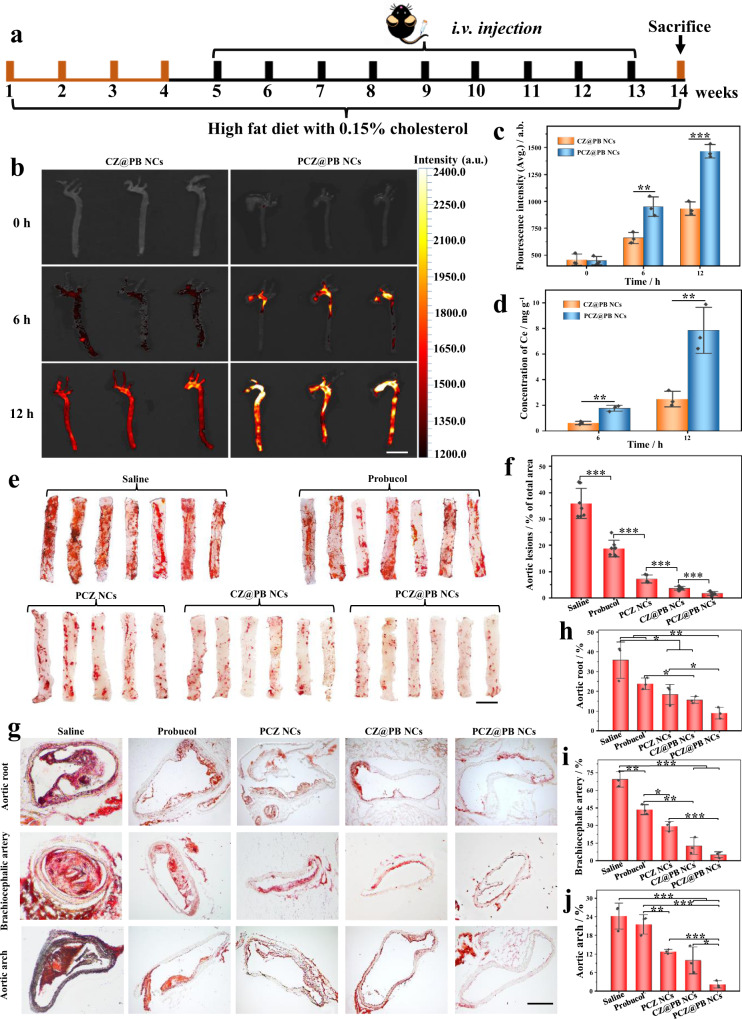
In vivo therapeutic effect of the biomimetic nanodrug, PCZ@PB NCs. **a** Schematic illustrating the treatment protocols. **b** Fluorescence images of CZ@PB NCs and PCZ@PB NCs accumulation in the aortas of atherosclerotic model mice at 0, 6, and 12 h. Scale bars, 50 mm. **c** Quantification of the fluorescence intensity of CZ@PB NCs and PCZ@PB NCs that accumulated in the aortas of atherosclerotic model mice at 0, 6, and 12 h, *n* = 3. **d** Inductively coupled plasma-mass spectrometry for quantifying cerium ion content in the aortas of atherosclerotic model mice treated with CZ@PB NCs and PCZ@PB NCs at 12 h, *n* = 3. **e** Representative photographs of oil red O (ORO)-stained aortas from mice following treatment with different formulations. Scale bars, 50 mm. **f** Quantitative analysis of the lesion area in aortas. *n* = 7 lesion area in aortas of saline and probucol treated mice. *n* = 6 lesion area in aortas of CZ@PB NCs and PCZ@PB NCs-treated mice. *n* = 5 lesion area in aortas of PCZ NCs-treated mice. **g**–**j** ORO-stained cryosections of the aortic root, aortic arch, and brachiocephalic artery. Scale bars, 200 μm. Quantitative analysis of the relative plaque area in sections of the aortic root (**h**), brachiocephalic artery (**i**), and aortic arch (**j**), *n* = 3. Data were presented as mean ± s.d. Analysis of mean fluorescence intensity, cerium ion concentration, and aortic lesion area was performed using one-way ANOVA. *P ≤ 0.05, **P ≤ 0.01, and ***P ≤ 0.001. Specific *P*-values are shown in the source data. Source data are provided as source data files.

The in vivo pharmacokinetic profile of PCZ@PB NCs was examined in mice (Supplementary Fig. 26). Following the intravenous (i.v.) injection of the fluorescent dye-labeled PCZ@PB NCs (IR 780-PCZ@PB NCs) were injected intravenously, the whole blood of mice was collected at corresponding timepoints, and anticoagulation was performed. The blood sample was diluted 5000 times using PBS, and the fluorescence intensity was quantified. The results showed that 71% of the IR 780-PCZ@PB NCs were removed from the blood after 12 h. We chose AS as a typical inflammatory disease model to study and develop the specific therapeutic effects of the nanomedicine in vivo. The atherosclerotic model was established by feeding apolipoprotein E-deficient (ApoE^−/−^) mice a high-fat diet for 3 months (Fig. 5a).

IR 780-labeled PCZ@PB NCs were administered to study the in vivo targeting ability of the nanodrugs and whether they effectively exerted a therapeutic effect (Fig. 5b, c). 6 h following i.v. injection, the mouse aorta was isolated, and fluorescence was detected in the aortic arch and abdominal aorta. A significantly higher fluorescence distribution was observed at 12 h. Inductively coupled plasma-mass spectrometry was used to quantify the exact content of cerium ions in the aorta (Fig. 5d), and the results showed that more PCZ@PB NCs accumulated in the aorta than CZ@PB NCs attributable to the active targeting ability of the platelet membrane. The colocalization of green fluorescence with macrophages was observed after the administration of PCZ@780 NCs compared with the nanodrug-treated group without platelet membrane modifications (Supplementary Fig. 27). Conversely, although CZ@780 NCs also exhibited fluorescence in the plaque sites, it was relatively weak and overlapped less with the fluorescence of macrophages than PCZ@780 NCs. These results confirmed that PCZ@PB NCs administered by i.v. injection accumulate in atherosclerotic lesions. Supplementary Fig. 28 shows the accumulation of PCZ@PB NCs in the heart, liver, spleen, lung, and kidneys.

### In vivo treatment of AS with PCZ@PB NCs

We evaluated the anti-inflammatory and antioxidant activities of PCZ@PB NCs to treat AS. ApoE^−/−^ mice were assigned to five different groups after receiving a Western diet for 1 month. Different preparations were administered for 2 months while maintaining the Western diet (Fig. 5a). After 2 months, the entire aorta was separated, stained with ORO, and characterized. The saline group showed large ORO-positive areas and a significant inhibition of plaque formation due to free PB (Fig. 5e). Conversely, much lesser ORO-stained areas were observed in the aortas of mice treated with PCZ NCs, CZ@PB NCs, or PCZ@PB NCs. Further quantification showed significant differences among the groups treated with i.v. saline, PB, PCZ NCs, CZ@PB NCs, or PCZ@PB NCs (Fig. 5f). Consistent with this result, the ORO-stained frozen sections of the aortic root, aortic arch, and cephalic brachial arteries also revealed that the nanocomplexes inhibited plaque generation, with the most significant antiatherosclerotic activity observed for PCZ@PB NCs (Fig. 5g–j). These results suggest that i.v. injected PCZ@PB NCs effectively inhibit atherosclerotic plaque generation via the dual active–passive targeting of atherosclerotic lesions, exerting both enzyme-like activity of CZ NCs and antioxidant activity of the coassembled PB.

Histochemical analyses of aortic root sections were performed to examine the composition of atherosclerotic plaques (Supplementary Fig. 29). Hematoxylin and eosin (H&E) staining showed the area of the vascular plaque. The plaque area was significantly smaller in the PCZ@PB NCs group than in the saline group. Masson’s trichrome staining demonstrated higher collagen content around the plaques in the PCZ@PB NCs-treated group. In addition, anti-CD68 and MMP-9 antibody staining showed that treatment with PCZ@PB NCs effectively reduced macrophage infiltration and MMP-9 expression in plaques. Alpha-Smooth muscle actin (α-SMA) antibody staining showed substantial smooth muscle cell accumulation in the aortic roots of the PCZ@PB NCs group, which is presumed to be beneficial for atherogenesis. Altogether, PCZ@PB NCs recruit smooth muscle cells, reduce macrophage infiltration, inflammatory factor expression, and plaque formation, and inhibit the progression of AS.

### In vivo safety assessment

It is imperative to establish the safety of nanodrugs; hence, the possible side effects of PCZ@PB NCs following 2 months of treatment were evaluated. In the sections of all the treated groups, no significant damage was observed except for the saline group, whose the liver sections exhibited fatty vacuoles due to continuous feeding of high-fat diet(Supplementary Fig. 30). In addition, the complete blood count and clinical biochemical analysis showed values within the normal range for each index (Supplementary Fig. 31). Therefore, these results suggest that i.v. PCZ@PB NCs administration is safe at the doses tested in our study.

## Discussion

Currently, AS treatment, a typical chronic inflammatory disease, relies primarily on drug therapy; however, and the low utilization and side effects are important reasons for poor treatment effecacy of such therapy^36,37^. Recently, the emergence of nanomaterials with ROS-scavenging abilities has opened an avenue for AS treatment^10,38,39^. Among them, ceria nanomaterials are rich in multienzymatic activities owing to their surface valence coexistence, which potentially facilitates ROS-scavenging and anti-inflammatory activities without redundant modifications. Further, ceria nanomaterials have been widely used to treat central and neurodegenerative diseases. However, only few applications of ceria nanomaterials are available for AS treatment because of their nonspecific distribution and poor degradability. Furthermore, the therapeutic effect of ceria nanozymes is restricted by their weak delivery capability, and the large size of Ce-MOF impairs their enzymatic activity. Therefore, developing a ceria nanomaterial with good delivery capability and multienzyme activity maintenance is key to expanding its biological applications.

In this study, ligands containing bisphosphonate and imidazole groups were self-assembled with cerium ions, generating new ceria-MOF-like nanomaterials (Supplementary Figs. 1–4). Multifunctional CZ NCs with a homogeneous size and multiple enzyme-mimetic activities were obtained using the innovative self-assembled small molecule, ZOL, as a linker (Fig. 2). These CZ NCs can also be used as delivery platforms for a variety of functional dye molecules and drugs via simple coassembly and may be a promising platform for the treatment of other diseases (Supplementary Fig. 12 and Supplementary Table 2). In addition, ZOL self-assembles with a variety of metal ions (Supplementary Fig. 7). Therefore, ZOL-metal ion-based self-assembly strategies may serve as a universal approach in various fields.

The coassembly of CZ NCs with PB, an atherosclerotic antioxidant drug, resolved the issue of low utilization of the latter due to its insolubility. Platelet membranes exhibiting a high affinity for inflammatory lesions were selected for the biomimetic treatment. The obtained PCZ@PB NCs not only inherited the multienzyme activity of the ceria nanozymes (Fig. 3) but were also efficiently taken up by RAW 264.7 cells (especially inflammatory RAW 264.7 cells) (Fig. 4a–d). PCZ@PB NCs scavenged the excess intracellular ROS (Fig. 4e–h and Supplementary Figs. 20–25), downregulated the expression of proinflammatory factor (Fig. 4i–l), and demonstrated synergistic inhibition with drugs on foam cell formation (Fig. 4m, n). These results suggest the potential of biomimetic PCZ@PB NCs for treating AS. In vivo experiments significantly increased the accumulation of platelet-modified PCZ@PB NCs in the aortas of ApoE^−/−^ mice (Fig. 5b–d and Supplementary Fig. 27). The atherosclerotic lesion area was smaller in mice treated with a tail vein injection and visible with both longitudinal and latitudinal arterial staining (Fig. 5e–j). Moreover, the H&E staining of the major organ sections and typical hematological parameters confirmed the good safety profile of PCZ@PB NCs (Supplementary Figs. 30, 31).

In conclusion, we propose a new strategy for assembling a specific small-molecule ligand, ZOL, with cerium ions to obtain multifunctional CZ NCs having ROS-scavenging, anti-inflammatory and drug transport capabilities. Based on this strategy, the platelet membrane-modified nanodrugs, PCZ@PB NCs, were developed as a bionic nanozyme platform for treating AS. Compared to conventional drug delivery platforms for atherosclerosis treatment (liposomes, silica, cyclodextrins, etc.)^16,40–42^, firstly, the assembly of CZ NCs with PB is achieved in a simple step, which not only solved the problem of low utilization caused by the insolubility of PB, but also effectively maintained the drug activity during storage and transport by shielding it from environmental oxidizing factors through its own enzymatic activity, such as oxygen in air. Secondly, compared to traditional passive drug delivery, platelet mimetics not only achieve active and passive targeting to the AS microenvironment, but also selective accumulation to inflammatory macrophages, which is conducive to the precise treatment of AS. Finally, in contrast to single drug delivery systems, PCZ@PB NCs as carriers also have a therapeutic effect on AS and can synergise the anti-AS effect of drugs. This nanozyme platform provides prospects for expanding the biomedical applications of ceria nanomaterials and broadens the potential for synergistic treatment of other inflammatory diseases, either alone or through further drug delivery.

## Methods

### Chemicals and materials

Cerium(III) nitrate hexahydrate (Ce(NO_3_)_3_·6H_2_O), alendronate sodium (AL), Tris(2,2-bipyridine) ruthenium chloride (RDPP), 2-methylimidazole (HMIM), riboflavin, rhodamine B, H_2_O_2_ (technical grade, 30%), LPS, and ORO were provided by Sigma–Aldrich (USA). Zoledronic acid hydrate (ZOL) was obtained from Damas-beta. PB and nitrotetrazolium blue chloride (NBT) were purchased from TCI (Japan). The CCK-8 kit was purchased from MCE (USA). oxLDL was purchased from Yiyuan Biotech (China). Chlorin e6 (Ce6) was provided by Frontier Scientific. A BCA protein concentration determination kit and a reactive oxygen detection kit were obtained from Beyotime Biotechnology. Penicillin–streptomycin solution (100X), fetal bovine serum (FBS), trypsin, Dulbecco’s modified Eagle’s medium (DMEM) and RPMI 1640 medium (1640) were obtained from Gibco (USA). High-purity (Milli-Q) water with a resistivity of 18.2 MΩ cm was obtained from an inline Millipore RiOs/Origin water purification system.

### Instruments

The following instruments were used: an ultrasonic cleaner (42 kHz, 100 W, Kun Shan Ultrasonic Instruments), an AFM (SHIMADZU), an HPLC system (Agilent Technologies), a multifunctional microplate inspection system (Thermo Fisher), a DLS particle size analyser (Malvern), a UV–vis absorption spectrophotometer (UV1050, Techcomp). a Nicolet iS5 instrument (Thermo Fisher Scientific, USA) for Fourier transform infrared (FT-IR) spectroscopy, an InVivo Elite Preclinical Optical Imaging System (InVivo Smart, VIEWORKS), a field emission TEM (Thermo Fisher), an SU8020 (HITACHI, Japan) for field emission SEM, an Agilent 7500ce ICP–MS (USA), a Nikon Eclipse Ti-S inverted fluorescence microscope (Nikon Corporation, Japan), an upright microscope (Nikon ECLIPSE Ci, Nikon Corporation), a fully automatic chemiluminescence fluorescence image analysis system (Tanon 5200, Shanghai Tanon), and a portable dissolved oxygen analyser (JPBJ-608, Shanghai INESA).

### Synthesis of dual-ligand nanocomposites Ce@HMIM@AL (CHA)

1. The aqueous solution of 100 μL of 0.1 M Ce(NO_3_)_3_·6H_2_O was slowly added dropwise to 1 mL of the AL aqueous solution (10 mg/mL). Then, 1 mL of HMIM aqueous solution (10 mg/mL) was added, and stirring was continued for 2 h to complete the reaction. Finally, the product was purified by dialysis with ultrapure water for 24 h.
2. One millilitre of an aqueous AL solution (10 mg/mL) and aqueous HMIM solution (10 mg/mL) were well mixed. Then, 100 μL of the prepared Ce(NO_3_)_3_·6H_2_O aqueous solution (0.1 M) were slowly added dropwise to the mixed system of AL and HMIM, and the reaction continued for 2 h. Finally, the product was purified by dialysis with ultrapure water for 24 h.
3. The aqueous solution of 100 μL of 0.1 M Ce(NO_3_)_3_·6H_2_O was added dropwise to 1 mL of HMIM aqueous solution (10 mg/mL). Then, 1 mL of the aqueous AL solution (10 mg/mL) was added, and stirring was continued for 2 h to allow a complete reaction. Finally, the product was purified by dialysis with ultrapure water for 24 h.
4. The aqueous solution of 100 μL of 0.1 M Ce(NO_3_)_3_·6H_2_O was slowly added dropwise to 1 mL of HMIM aqueous solution (10 mg/mL), and then 1 mL of ultrapure water was added and stirred for 2 h to allow the complete reaction. Finally, the product was purified by dialysis with ultrapure water for 24 h.
5. The aqueous solution of 100 μL of 0.1 M Ce(NO_3_)_3_·6H_2_O was added dropwise to 1 mL of AL aqueous solution (10 mg/mL). Then, 1 mL of ultrapure water was added, and the stirring was continued for 2 h to allow the complete reaction. Finally, the product was purified by dialysis with ultrapure water for 24 h.
6. First, 200 μL of Tris pH 8.8 buffer were added to 1.8 mL of ultrapure water to reach pH 8.8. Then, 100 μL of a 0.1 M Ce(NO_3_)_3_·6H_2_O aqueous solution were slowly added dropwise to the buffer solution, and stirring was continued for 2 h to fully react. Finally, the product was purified by dialysis with ultrapure water for 24 h.
7. First, 100 μL of 0.1 M Ce(NO_3_)_3_·6H_2_O aqueous solution were slowly added dropwise to 1 mL of the aqueous AL solution (10 mg/mL). Then, 1 mL of ultrapure water (pH 8.8) was added, and stirring was continued for 2 h to allow complete reaction. Finally, the product was purified by dialysis with ultrapure water for 24 h.

The obtained samples were characterized by capturing photographs and performing AFM, SEM, or TEM.

### CHA delivery capability evaluation

The delivery ability of CHA may be evaluated by determining whether CHA coassembles with dye molecules or drug molecules such as PB to form nanoparticles. Briefly, adding dye or PB to react based on Ce, AL, and HMIM self-assembly is a simple one-pot method. First, the hydrophobic molecules were dissolved in DMSO, and the hydrophilic molecules were dissolved in ultrapure water to obtain a solution with a concentration of 10 mM. Then, 10 mg of AL and 10 mg of HMIM were each completely dissolved in 1 mL of ultrapure water, transferred to the reaction flask and mixed well. Then, 100 μL of the aqueous Ce(NO_3_)_3_·6H_2_O solution (0.1 M) were added slowly. Finally, 10 μL of 10 mM solutions of various molecules were added and stirred for 2 h. The product obtained from the reaction of hydrophobic molecules was centrifuged at 5000 rpm (1740 × *g*) for 5 min in a sleeve centrifuge tube (10 kDa MWCO) and washed twice with ultrapure water for purification, and the volume was adjusted to 1 mL. The reaction product involving hydrophilic molecules was purified by dialysis against ultrapure water for 24 h. After CHA and dye were coassembled, UV–vis spectroscopy was used to confirm the presence of the dye. After CHA and PB were assembled, the morphology was characterized using TEM.

### General investigation of the ZOL-metal ion self-assembly strategy

The generalizability of the strategy was assessed by examining the self-assembly of ZOL with different metal ions. For this purpose, 50 μL of various metal ion solutions (0.1 M, Ce^3+^, Ca^2+^, Zn^2+^, Mn^2+^, Fe^3+^, and Cu^2+^) were slowly added dropwise to 1 mL of ZOL (10 mg/mL), and the reaction was allowed to proceed for 2 h. The products were collected and dialyzed against pure water for 24 h for purification, and the particle size was characterized using DLS.

### Synthesis of single-ligand nanocomposites Ce@ZOL (CZ NCs)

ZOL was selected as the linker to be coassembled with the cerium ion in the reaction. Briefly, 10 mg of ZOL were dispersed in 1 mL of ultrapure water containing 10% Tris pH 8.8, fully dissolved and transferred to the reaction flask. Subsequently, 50 μL of the aqueous Ce(NO_3_)_3_·6H_2_O solution (0.1 M) were slowly added dropwise and stirred for 2 h. Finally, the collected product was dialyzed against ultrapure water for 24 h for purification. The synthesis of CZ NCs was characterized using TEM, AFM, and IR spectroscopy.

### Evaluation of the CZ NCs delivery capability

The coassembly capability of CZ NCs was evaluated by determining the formation of nanoparticles with dyes or drugs. Briefly, dyes or drugs were added based on the coassembly of Ce and ZOL to react together in a simple one-pot method. First, the hydrophobic molecules were dissolved in DMSO, and the hydrophilic molecules were dissolved in ultrapure water to obtain 10 mM solutions. Then, 10 mg of ZOL were dispersed in 1 mL of ultrapure water containing 10% Tris pH 8.8 and transferred to the reaction flask after being completely dissolved. Then, 50 μL of an aqueous Ce(NO_3_)_3_·6H_2_O solution (0.1 M) were added slowly dropwise. Finally, 10 μL of the prepared 10 mM solution of various molecules were added, and stirring was continued for 2 h. The products obtained from the reaction containing hydrophobic molecules were centrifuged at 5000 rpm (1740 × *g*) for 5 min in a sleeve centrifuge tube (10 kDa MWCO), washed twice with ultrapure water for purification, and the volume was adjusted to 1 mL. The products of the reaction involving hydrophilic molecules were purified by dialysis with ultrapure water for 24 h. After CZ NCs were coassembled with the dyes, UV–Vis spectroscopy was used to verify the presence of the dyes. After CZ NCs and drug molecules were coassembled, the hydrated particle size of the products was characterized.

### Synthesis of CZ NCs and PB coassembled nanocomposites (CZ@PB NCs)

The nanocomposite of CZ NCs and the drug PB (CZ@PB NCs) was coassembled using a one-pot method. Ten milligrams of ZOL were dispersed in 1 mL of ultrapure water containing 10% Tris pH 8.8 and transferred to a reaction flask after being fully dissolved. Then, 50 μL of the aqueous Ce(NO_3_)_3_·6H_2_O solution (0.1 M) and 30 μL of the PB solution (10 mM, DMSO) were added dropwise. After reacting for 2 h, the product was collected in a sleeve centrifuge tube (10 kDa MWCO), centrifuged at 5000 rpm (1740 × *g*) for 5 min, and washed twice with ultrapure water, and the volume was adjusted to 1 ml. The synthesis of CZ@PB NCs was characterized using TEM, AFM and UV spectroscopy.

### Quantitation of the PB concentration

HPLC was chosen to detect the concentration of PB. The first step was to configure the standard solution. PB standards were accurately weighed and dissolved in methanol to obtain a 500 μg/mL PB stock solution. A certain volume of the PB stock solution was precisely measured in a volumetric flask and diluted with methanol to obtain a series of standard solutions with different concentrations (0.1, 0.2, 0.5, 1, 2.5, 10, 20, and 30 μg/mL). Then, 50 μL of ultrapure water and 50 µL of the PB solution from the series were mixed to obtain a series of samples with concentrations of 0, 0.05, 0.1, 0.25, 0.5, 1.25, 5, 10, and 15 μg/mL. Then, 300 µL of methanol were added to the sample, and the sample was analyzed after sufficient mixing. The ordinate represents the peak area of PB (A), and the abscissa represents the concentration of PB (ρ, μg/mL). The standard curve was drawn, and the minimum detection limit was calculated. Finally, the sample was processed and tested. Fifty microlitres of ultrapure water, 50 µL of sample and 300 µL of methanol were mixed and injected for determination.

Chromatography conditions: The column type was Shim-pack VP-ODs (4.6 × 150 L, SHIMADZU), and methanol-water (95:5, V/V) was used as the mobile phase. The injection volume was 20 μL, and the flow rate was 1.0 mL/min. Chromatographic information was acquired at a wavelength of 242 nm at room temperature.

### Synthesis of biomimetic nanodrug platelet membrane-coated CZ@PB NCs (PCZ@PB NCs)

The platelet membrane was obtained using the method reported in the literature^43,44^. Briefly, blood was collected from mice, anticoagulated, mixed gently and centrifuged (200×g) for 15 min to obtain the upper layer of platelet-rich plasma (PRP). Then, the platelets were precipitated by centrifugation at 800 × *g* for 20 min, and the supernatant was discarded. The platelets were resuspended in PBS containing 1 mM EDTA, freeze-dried at −80 °C, thawed at room temperature, and precipitated by centrifugation at 4000 × *g* for 3 min. After three washes with the washing solution, platelet membrane vesicles were obtained. The platelet membrane vesicles were suspended in water and sonicated for 5 min, mixed with CZ@PB NCs and sonicated again for 2 min, then removed and placed at 4 °C for 30 min. A subsequent centrifugation step was performed at 3000 × *g* for 30 min to remove the uncoated membrane, and the sample was redispersed in ultrapure water to complete the platelet membrane coverage. The synthesis of CZ@PB NCs was characterized by performing uranyl acetate negative staining and TEM, DLS, and zeta potential measurements, among other tests.

### Verification of the platelet membrane coating

The platelet membrane coating was verified using sodium dodecyl sulfate–polyacrylamide gel electrophoresis (SDS–PAGE). As previously reported, platelet membrane vesicles and PCZ@PB NCs were lysed with radio immunoprecipitation assay (RIPA) lysis buffer, and the total protein contents in the samples were quantified using a Pierce BCA protein assay kit. Samples mixed with SDS–PAGE sample loading buffer were heated at 100 °C for 10 min. Then, samples with equivalent protein amounts (40 μg/well) were loaded on a 6% SDS–PAGE gel and run at 120 V for 2 h. The proteins in the gel were stained with Coomassie Blue for 2 h and washed overnight for subsequent imaging.

### Verification of the H2O2-scavenging capability

The removal of H_2_O_2_ by CZ NCs, CZ@PB NCs and PCZ@PB NCs was accompanied by the production of oxygen. Therefore, the oxygen content in the solution was measured using a dissolved oxygen metre. One millilitre of CZ NCs was transferred to a glass reaction flask, and different concentrations of H_2_O_2_ were added with continuous stirring (200 rpm, 70 g) to prepare a concentration gradient of 0, 50, 100, 200, 400, 800, 1600, and 3200 mM H_2_O_2_. The oxygen concentration in the solution was determined after 3 min.

### Determination of the superoxide dismutase-like activity of PCZ@PB NCs

The SOD activity of the nanodrug PCZ@PB NCs was studied using a reported method^33^. Riboflavin (100 μL, 1.2 mMol/L) was added to 150 μL of nitrated blue tetrazolium chloride (2 mMol/L), 400 μL of EDTA (0.1 mol/L), and 5.8 mL of sodium phosphate buffer (pH 7.8, 10.0 mMol/L), and was used as a detection solution. Fifty microlitre aliquots of different samples (PB, platelet membranes, CZ@PB NCs, and PCZ@PB NCs, 2 μM) were mixed with 100 μL of detection solution (*n* = 3). The mixture was shaken at 37 °C for 5 min and then irradiated with a 27 W light tube for 2 min to measure the absorbance at 560 nm.

### Blood compatibility

Whole blood was collected from mice and used to prepare a 5% red blood cell suspension. The PBS group served as a negative control, and the sodium dodecyl sulfate group was used as a positive control. At the same time, different concentrations of PCZ@PB NCs were mixed with 5% red blood cells and incubated for 24 h. The upper suspension was aspirated, and the degree of erythrocyte rupture was evaluated using UV–Vis spectroscopy.

### Cell safety

The cell safety of CZ NCs, CZ@PB NCs, and PCZ@PB NCs was assessed using the CCK-8 cell viability kit assay. Briefly, HUVECs and mouse macrophage RAW 264.7 cells were seeded in a 96-well plate at densities of 4000 and 5000 cells per well, respectively, and cultured for 24 h. The viability of blank wells containing medium alone in each plate was defined as 0%, and the wells of PBS-treated cells were defined as 100% viability. The cells were then exposed to a series of concentrations of CZ NCs, CZ@PB NCs, and PCZ@PB NCs for 24 h. Subsequently, the cells were washed thoroughly with PBS, and the CCK-8 solution was added to each well to a concentration of 10%. The plate was incubated at 37 °C, and the absorbance was measured at 450 nm.

### Cell uptake study

First, the uptake of PCZ@PB NCs in macrophages was studied. RAW 264.7 cells were inoculated into confocal dishes and cultured overnight to allow them to adhere to the well. After 8 h of PCZ@PB NC treatment, the cells were stained with DAPI and a lysosome tracer and characterized using confocal microscopy.

Next, the uptake of CZ@PB NCs and PCZ@PB NCs in normal and inflammatory RAW 264.7 cells was studied. RAW 264.7 cells were seeded in two 12-well plates at a density of 50,000 cells per well and incubated for 24 h. One plate was cultured to maintain RAW 264.7 cells, and RAW 264.7 cells in the other plate was induced to form foam cells; for this experiment, the cells were treated with LPS (100 ng/ml) for 24 h. PBS, CZ@PB NCs, and PCZ@PB NCs were incubated with RAW 264.7 cells or foam cells for 8 h. After rinsing each well with PBS three times, the cells were collected and tested using flow cytometry (*n* = 3).

Finally, the uptake of CZ@PB NCs and PCZ@PB NCs within 24 h was studied. RAW 264.7 cells were seeded into a 24-well plate at a density of 25,000 cells per well and cultured overnight to allow them to adhere to the well. CZ@PB NCs (IR 780) and PCZ@PB NCs (IR 780) were added to each well at different times, and the cells in each well were uniformly washed with PBS, scraped and transferred to 1.5 mL EP tubes and centrifuged at 1000 rpm (350 × *g*) for 3 min. The cells were then resuspended in 200 μL of PBS, and their fluorescence intensity was measured using flow cytometry (*n* = 3).

### ROS-scavenging and anti-inflammatory activities of PCZ@PB NCs in macrophages

The levels of H_2_O_2_ and LPS-induced inflammation in the cells were measured using a reactive oxygen detection kit. The normal group was treated with fresh medium, and the model group was stimulated with 400 μM H_2_O_2_ or 100 ng/mL LPS. RAW 264.7 cells were treated with different concentrations of PCZ@PB NCs combined with the DCFH-DA probe and incubated for 30 min. Finally, fluorescence images were captured using a fluorescence microscope, and the fluorescence intensity was quantified using flow cytometry.

A western blot (WB) assay was selected to evaluate the anti-inflammatory effects of PCZ@PB NCs on macrophages in vitro. Specifically, RAW 264.7 cells were seeded in a 6-well plate at a density of 1 × 10^5^ cells per well. After 12 h, the normal group was treated with fresh medium, and the model group was stimulated with 100 ng/mL LPS and 100 IU/mL IFN-γ. The other groups were preincubated with CZ@PB NCs or PCZ@PB NCs for 2 h and then costimulated with 100 ng/mL LPS and 100 IU/mL IFN-γ for 24 h. RAW 264.7 cells were collected after different treatments, and intracellular proteins were extracted with RIPA lysis buffer on ice. The protein concentration was determined using a bicinchoninic acid protein detection kit (Biosharp, China). The proteins were separated by sodium dodecyl sulfate–polyacrylamide gel electrophoresis and transferred to a polyvinylidene fluoride membrane. After blocking with 5% BSA in TBST for 2 h, the membrane was incubated overnight with an anti-MMP-9 antibody (ab38898, 1:1000, abcam), anti-TNF-α antibody (ab183218, 1:1000, abcam), anti-NF-κB antibody (ab76302, 1:1000, abcam), and anti-GAPDH antibody (AF0006, 1:1000, Beyotime) on a shaker at 4 °C and then incubated for 2 h at room temperature with the corresponding secondary antibody. After three washes with TBST and an incubation with enhanced chemiluminescence reagent for 5 min, the membrane was visualized using a chemiluminescent imaging system (Tanon-5200, China).

### The effect of PCZ@PB NCs treatment on foam cell formation

RAW 264.7 cells were inoculated into a well plate at a density of 80,000 cells/well and cultured for 2 h. Then, the cells were treated with PB (5.2 μg/ml), CZ NCs (50 μM), CZ@PB NCs (50 μM), or PCZ@PB NCs (50 μM) and coincubated with oxLDL (100 μg/mL) for 48 h. The normal control group was treated with fresh medium, and the model group was stimulated with oxLDL alone. After three washes with PBS and fixation with 4% paraformaldehyde for 20 min, the cells were stained with 0.3% ORO. Finally, the cells were washed again with PBS three times and observed under an optical microscope.

### Animals

All animal experiments procedures and protocols were approved by the Experimental Animal Center of Chongqing Medical University (SCXK2018-0003). Male apolipoprotein E-deficient (ApoE^−/−^) mice (~6 weeks old) were supplied by the Ensiweier Biotechnology Co., Ltd. (China). ApoE^−/−^ mice were fed a high-cholesterol diet throughout the entire animal experiment.

### In vivo plaque targeting and tissue distribution of nanodrugs

Using IR 780-labelled CZ@PB NCs and PCZ@PB NCs, we examined the targeting ability of nanodrugs after an i.v. injection in ApoE^−/−^ mice. The CZ@PB NCs and PCZ@PB NCs were administered to mice at 80 mg/kg. Mice in the control group were treated with the same volume of saline. At predefined timepoints, the aortas and main organs were removed for ex vivo fluorescence imaging, and the MFI was analyzed using Living Image software. Furthermore, the imaged blood vessels were digested with concentrated nitric acid, and the concentration of cerium ions was quantified using ICP–MS.

### PCZ@PB NCs as a treatment for AS in vivo

ApoE^−/−^ mice were fed a high-fat diet for 3 months. After the first month, they were randomly assigned to five groups (*n* = 10), and different treatments were administered for an additional 2 months. Mice in the model control group were treated with saline, while animals in the other four groups were i.v. injected with free PB at 1.53 mg/kg, CZ NCs (80 mg/kg), CZ@PB NCs (80 mg/kg), and PCZ@PB NCs at 80 mg/kg. The 80 mg/kg nanocomposites contained a dose of PB equivalent to 1.53 mg/kg free PB. All formulations were i.v. injected twice per week.

### ORO staining of aortic plaques

After different treatments, the aortic diseased area was evaluated. After the ApoE^−/−^ mice were euthanized, the aorta was excised and perfused with PBS. The aorta was opened longitudinally and stained with ORO. In addition, frozen sections of the aortic root, aortic arch, and brachiocephalic artery were prepared and stained with ORO. The plaque area was calculated using ImageJ software.

### Histology and immunohistochemistry

After fixation with 4% paraformaldehyde, paraffin sections of aortic roots (5 μm thick) were prepared and stained with H&E and Masson’s trichrome. For the immunohistochemical analysis, 5 μm aortic tissue sections were incubated with antibodies against CD68, MMP-9, and α-SMA. ImageJ software was used for the semiquantitative analysis of histological images. The main organ sections were stained with H&E.

### In vivo safety assessment

Treated mice were anaesthetized, and blood was collected to verify the biocompatibility of PCZ@PB NCs. The blood samples were used for routine blood testing, the separated serum sample was used for biochemical analysis, and the main organs were used for H&E staining.

### Statistical analysis

All data are presented as the means ± standard deviations (SD). One-way ANOVA were utilized for statistical analyses. Values of *P ≤ 0.05, **P ≤ 0.01, and ***P ≤ 0.001 were applied to annotate statistical significance.

### Reporting summary

Further information on research design is available in the Nature Research Reporting Summary linked to this article.

## Supplementary information

Supplementary information

Description of Additional Supplementary Files

Supplementary Video 1

Supplementary Video 2

Reporting Summary

## Data Availability

All the data supporting the findings of this study are available within the article, source data and its supplementary information files. Supplementary Videos 1–2 are provided as Supplementary Video 1 and Supplementary Video 2, respectively, in the Supplementary Information file. Source data are provided with this paper.

## Source data

Source Data
