# Supplementary material for: Small molecule-assisted assembly of multifunctional ceria nanozymes for synergistic treatment of atherosclerosis
Source: Nat Commun. 2022 Nov 1;13:6528. doi: 10.1038/s41467-022-34248-y (PMC9626479; doi:10.1038/s41467-022-34248-y)
Supplement: Supplementary file 1 — Supplementary information [file 41467_2022_34248_MOESM1_ESM.pdf]

Supplementary Information

Small-molecule-assisted assembly of multifunctional ceria nanozymes for atherosclerosis synergistically treatment

Xiaoxue Fu[1], Xiaojuan Yu[1], Junhao Jiang[1], Jiaxin Yang[1], Lu Chen[1], Zhangyou Yang[1, *], Chao Yu[1, *]

1 Chongqing Key Laboratory for Pharmaceutical Metabolism Research, Chongqing pharmacodynamic evaluation engineering technology research center, College of Pharmacy, Chongqing Medical University, Chongqing 400016, P.R. China

Corresponding authors

E-mail: yangzhangyou@cqmu.edu.cn (Prof. ZY Yang.)

yuchao@cqmu.edu.cn (Prof. C Yu.)

Supplementary Figures 1–31

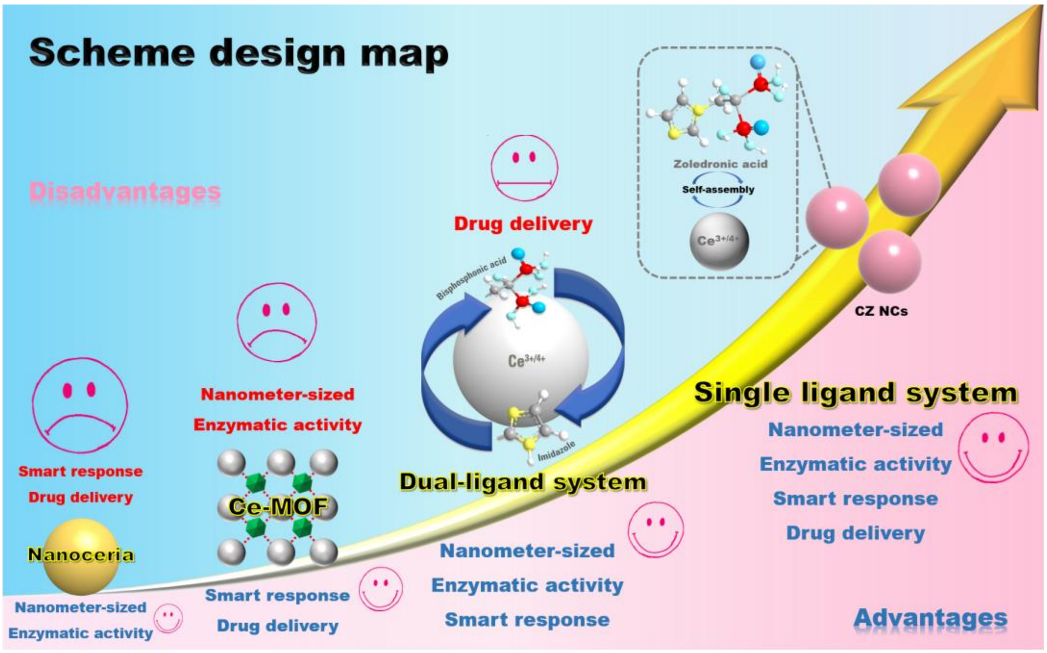

Supplementary Figure 1. Scheme design map. Typical advantages and disadvantages of existing different types of cerium-related nanomaterials. Ce-MOF represent the cerium metal organic frameworks. The dual-ligand system refers to the cerium nanocomposites synthesis system mediated by the two small molecule ligands, alendronic acid and dimethylimidazole, developed in this paper. The single-ligand system refers to the system proposed in this paper in which zoledronic acid is used as a linker to mediate the formation of MOF-Likely cerium nanocomposites.

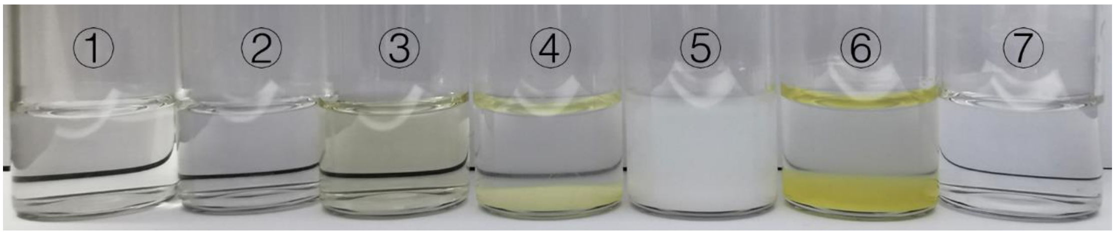

Supplementary Figure 2. Comparison photos of the products of the dual-ligand involved in the different synthesis conditions. In order to investigate the necessity of AL and HMIM acting together as dual-ligands, we controlled the experimental conditions. As shown in the pictures ①~③, even if the ingredients are added in a different order, only in the dual-ligand system, that is, when alendronic acid and dimethylimidazole coexist, the system exhibits a completely clarified and transparent state. But the color of the product is different, which indicates the possible difference in the product. Moreover, in the absence of alendronic acid and/or dimethylimidazole, the system (④~⑦) showed the characteristics of instability or visible precipitation.

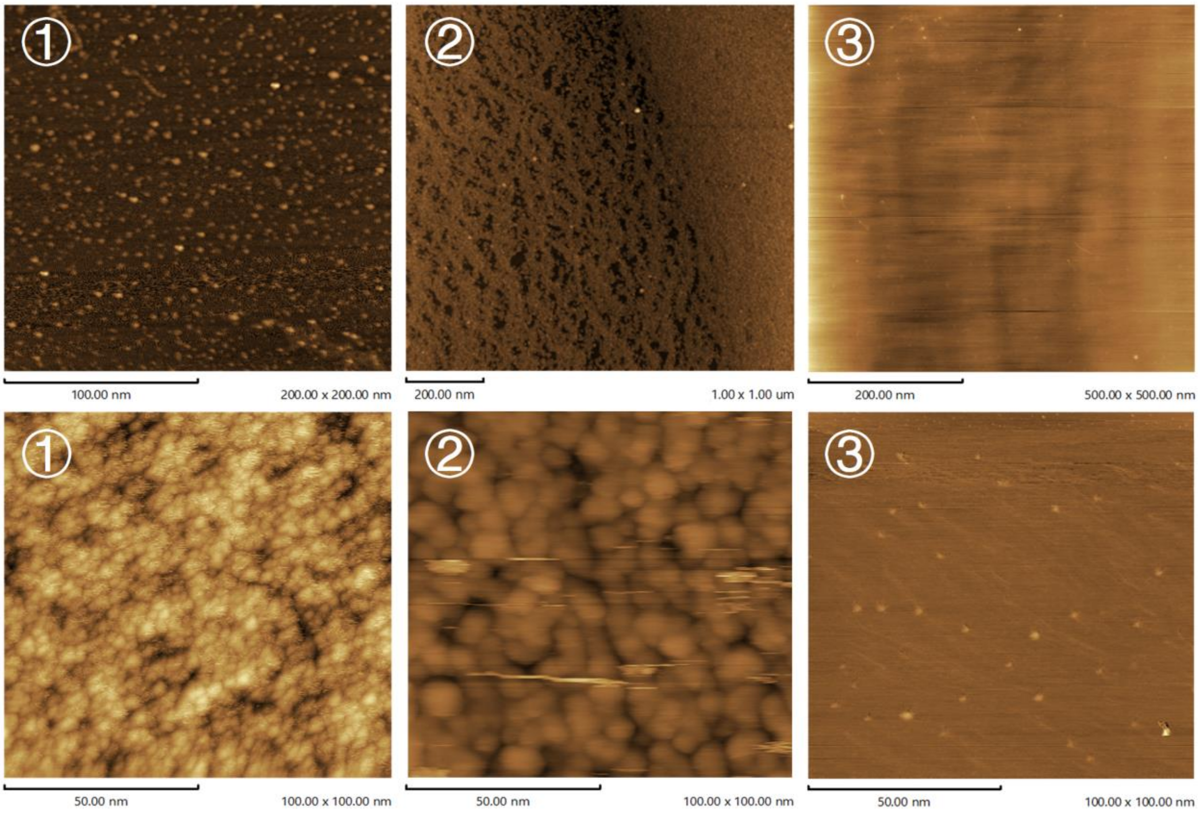

Supplementary Figure 3. AFM of the products of the dual-ligand involved in the different synthesis conditions. Samples ①~③ are products of dual-ligand system with different addition sequence of AL, HMIM and cerium nitrate. In order to further study the effect of the order of addition in the process, AFM was used to characterize the morphology of the product. The AFM results of ① show that the particle size of the product obtained by mixing AL and cerium ions and then adding HMIM is slightly larger than the reported AL-modified cerium oxide nanoparticles, but the homogeneity is poor. In contrast, once cerium nitrate comes into contact with alkaline dimethylimidazole first, it will form smaller-sized cerium oxide nanoparticles, and the later added AL only acts as a stabilizing effect on the outside (③). The AFM results of ② show that only in the neutral system where AL and HMIM are uniformly mixed, the addition of cerium ions can produce a uniformly dispersed nanocomposite that is significantly different from the current cerium oxide nanoparticles. Therefore, the above results indicate that the order of addition affects the product as a key factor, and the number ② as a dual-ligand system is more in line with our research expectations. The experiments were repeated for three times (n = 3) with similar results.

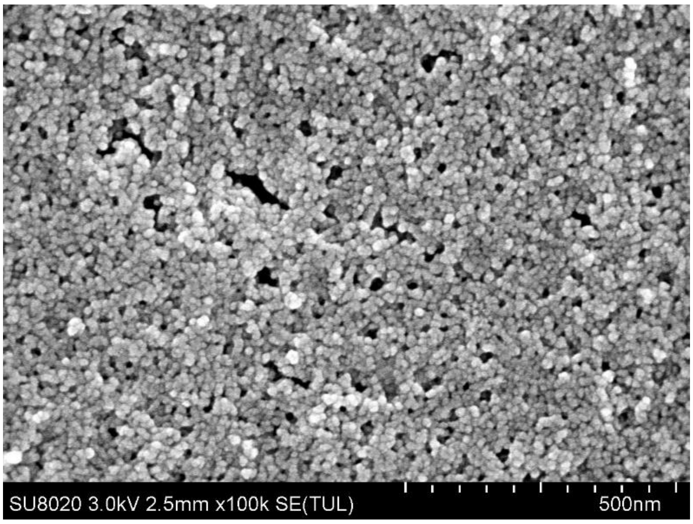

Supplementary Figure 4. SEM of dual-ligand ceria nanozymes platform (CHA). The experiments were repeated for three times (n = 3) with similar results.

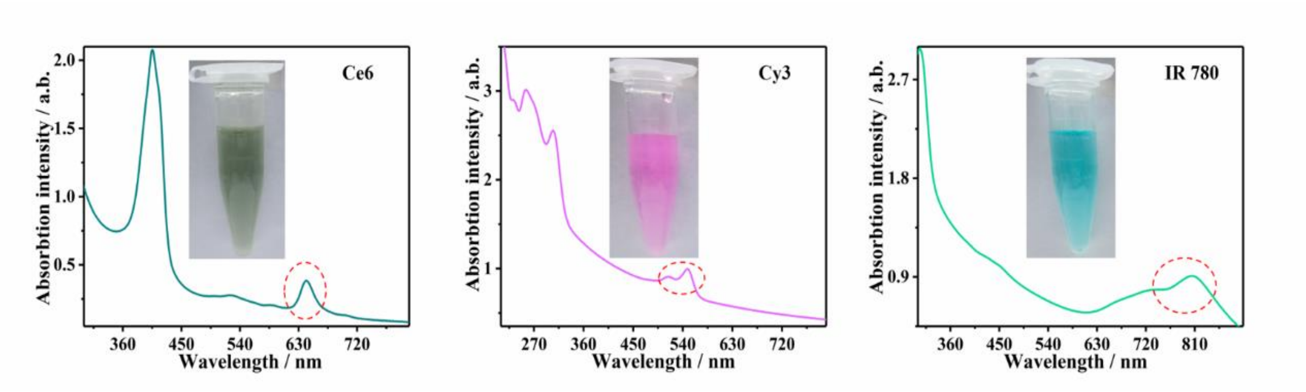

Supplementary Figure 5. Photographs and UV-Vis absorption spectra of the CHA assembled with dyes.

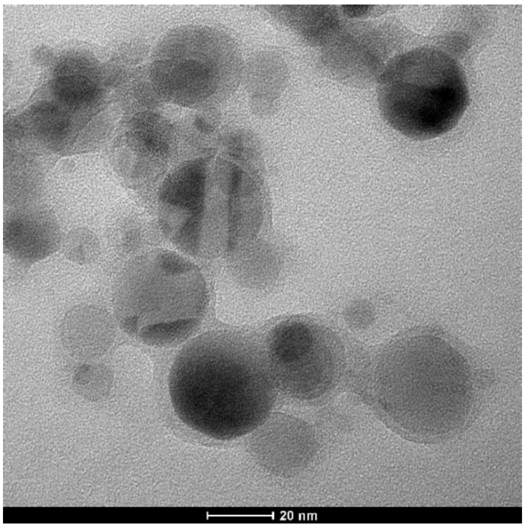

Supplementary Figure 6. TEM of the CHA assembled with the drug probucol. The particle size increased after the co-assembly of CHA with probucol. However, the homogeneity and stability of the products were affected by the relatively complex environment due to the co-participation of multiple molecules in the reaction mediated by dual ligands. The experiments were repeated for three times (n = 3) with similar results.

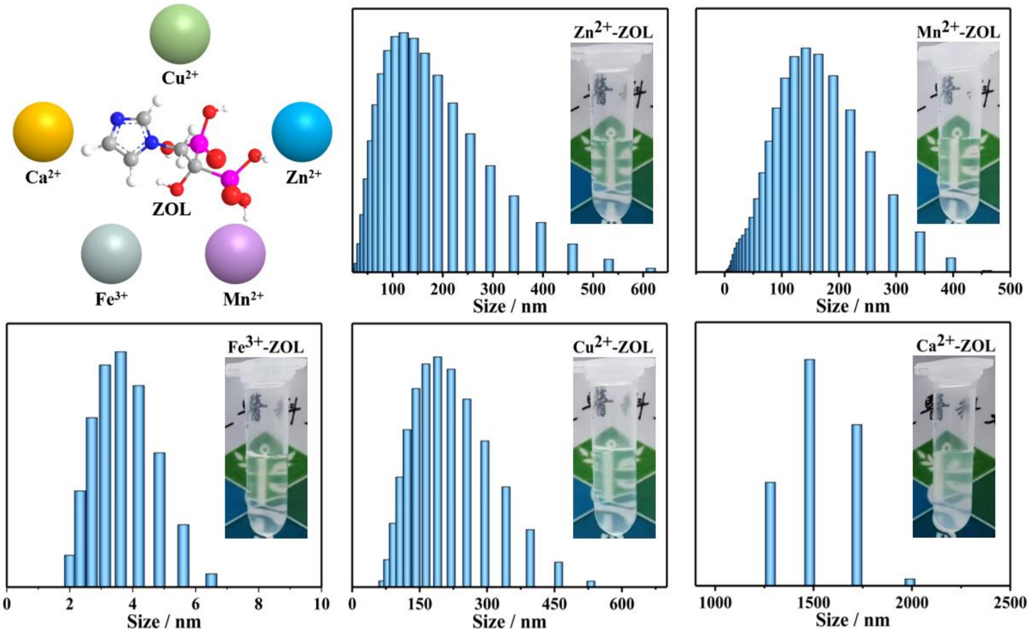

Supplementary Figure 7. DLS characterization of different metal ions and zoledronic acid (ZOL). The results show that ZOL can be co-assembled with Zn^{2+}, Mn^{2+}, Fe^{2+}, Cu^{2+}， Ca^{2+} to form a multi-metal nanocomposites.

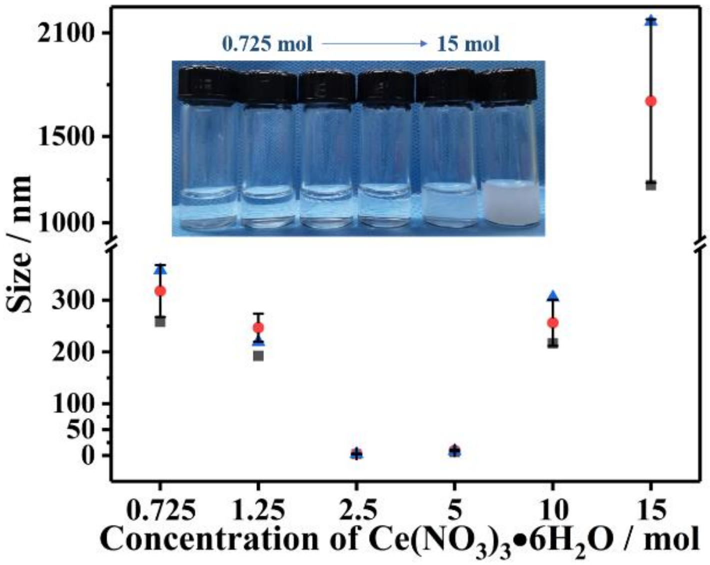

Supplementary Figure 8. Hydrated particle size of the reaction product after different concentrations of $Ce(NO_3)_3 \cdot 6H_2O$ mixed with 10mg/mL Zoledronic acid (ZOL) in the single-ligand system. The experiments were repeated for three times (n = 3) and data were presented as mean ± s.d.

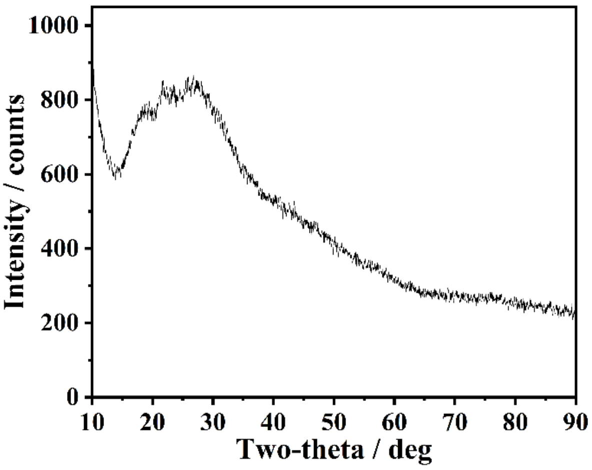

Supplementary Figure 9. XRD analysis of CZ NCs.

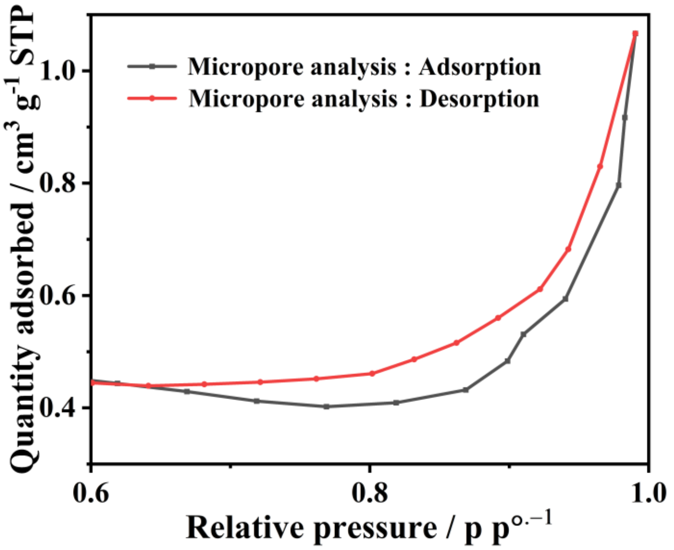

Supplementary Figure 10. Nitrogen adsorption and desorption curve of CZ NCs.

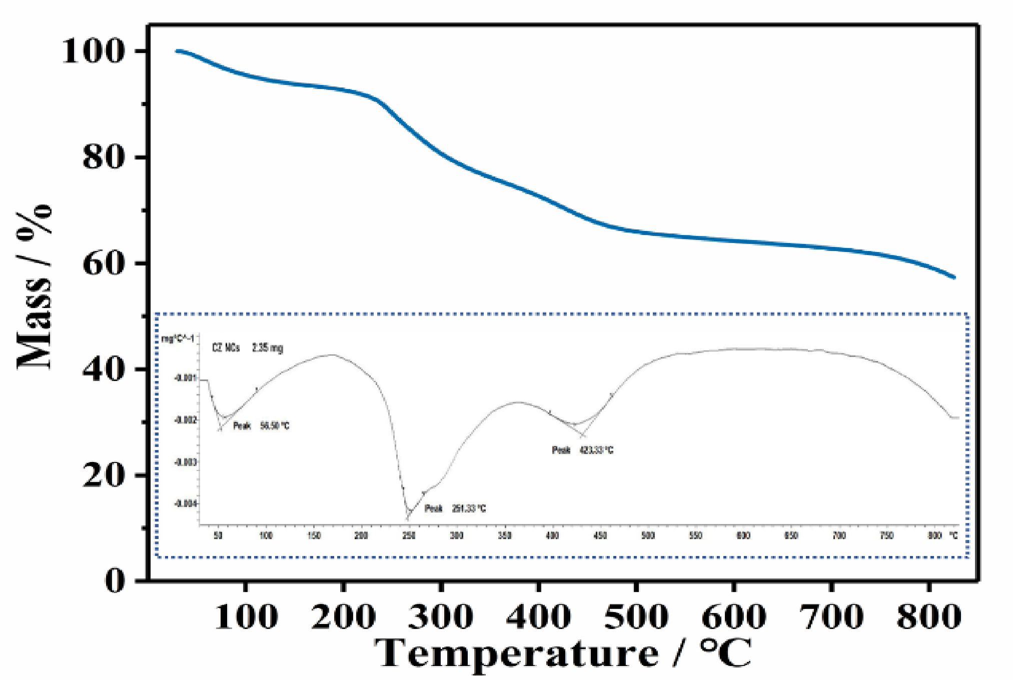

Supplementary Figure 11. Thermogravimetric analysis of CZ NCs.

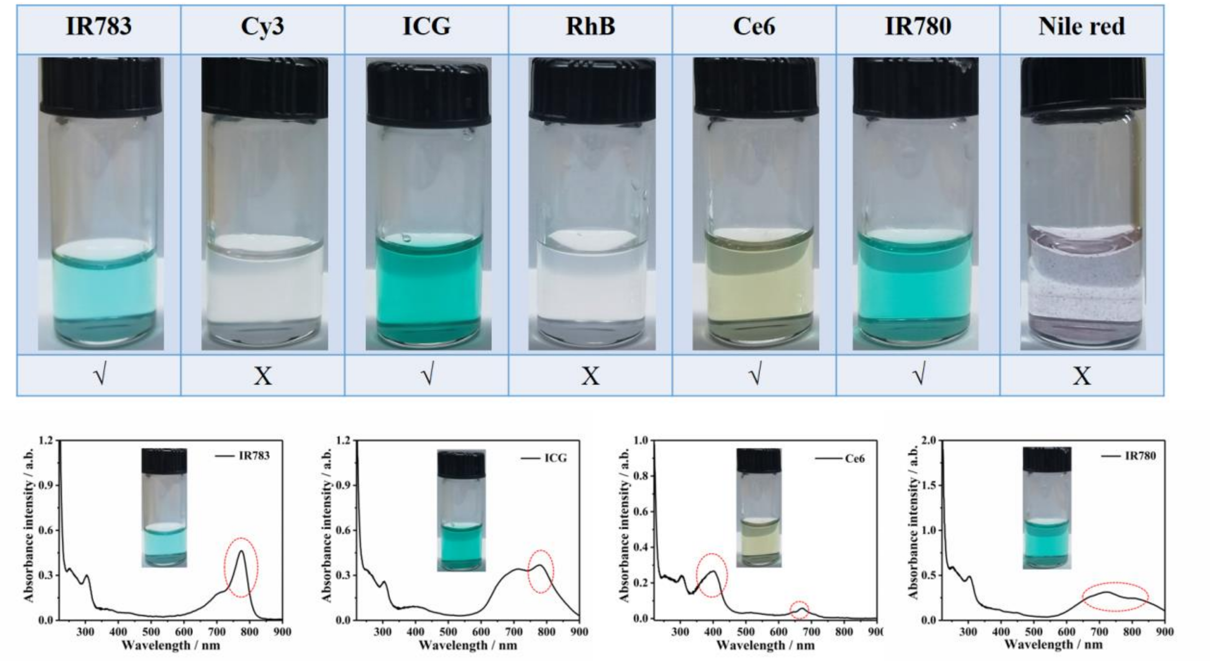

Supplementary Figure 12. Photographs and UV-Vis spectra of the purified products after the co-assembly of CZ NCs and different dyes.

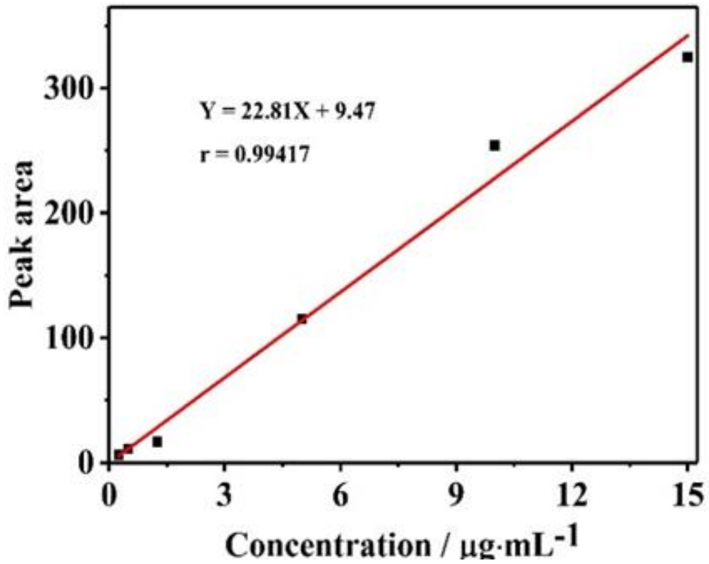

Supplementary Figure 13. Probucol concentration standard curve.

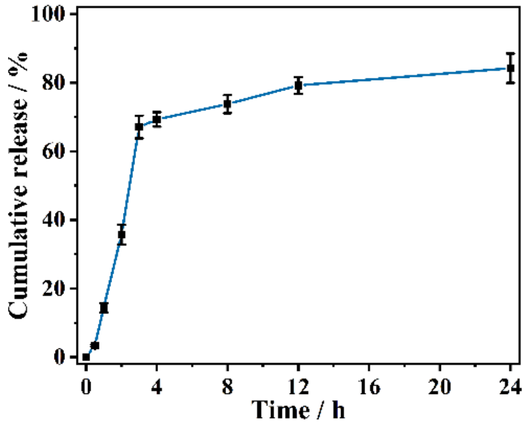

Supplementary Figure 14. PB release curve. The experiments were repeated for three times (n = 3) and data were presented as mean ± s.d.

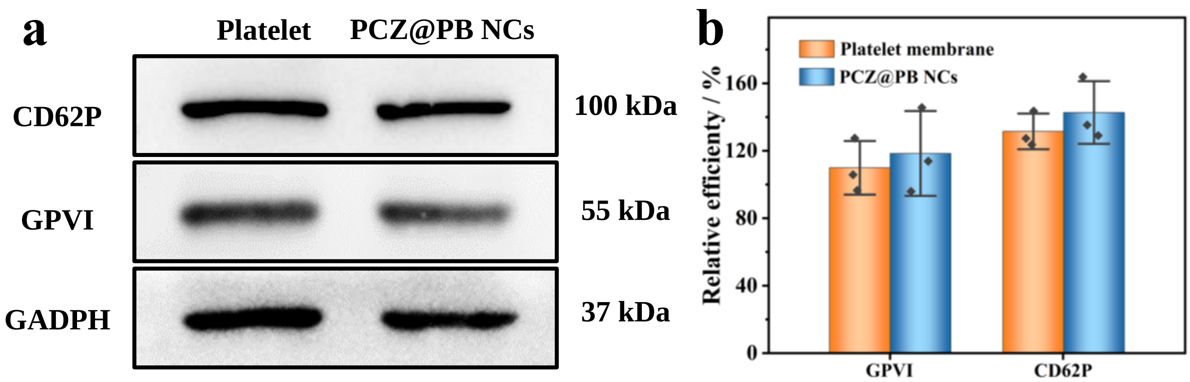

Supplementary Figure 15. Effects of PCZ@PB NCs on platelet activation. (a) WB analysis of platelet activation. (b) Quantification of GPVI and CD62P expression levels. The experiments were repeated for three times (n = 3) and data were presented as mean ± s.d.

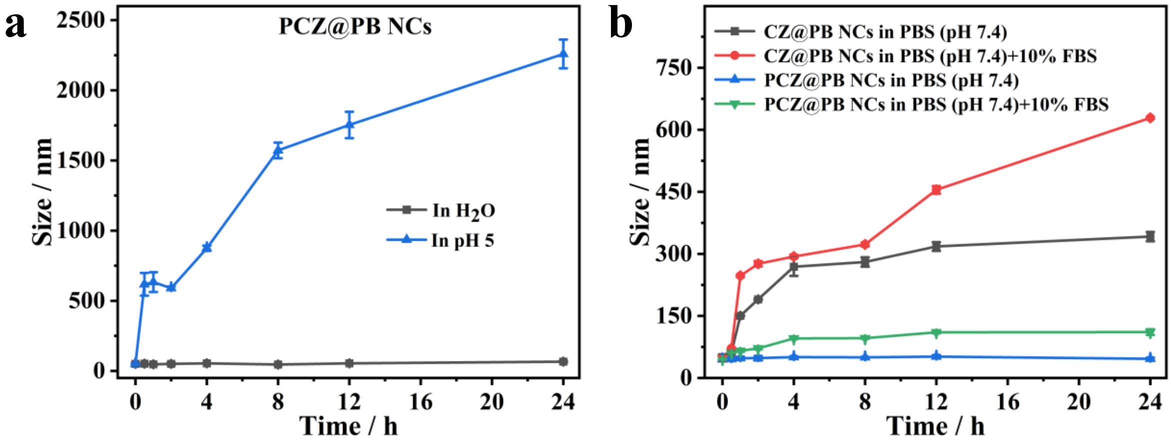

Supplementary Figure 16. a)Hydration particle size changes of PCZ@PB NCs in acidic environment. b) Particle size variation of CZ@PB NCs and PCZ@PB NCs in different media. The experiments were repeated for three times (n = 3) and data were presented as mean ± s.d.

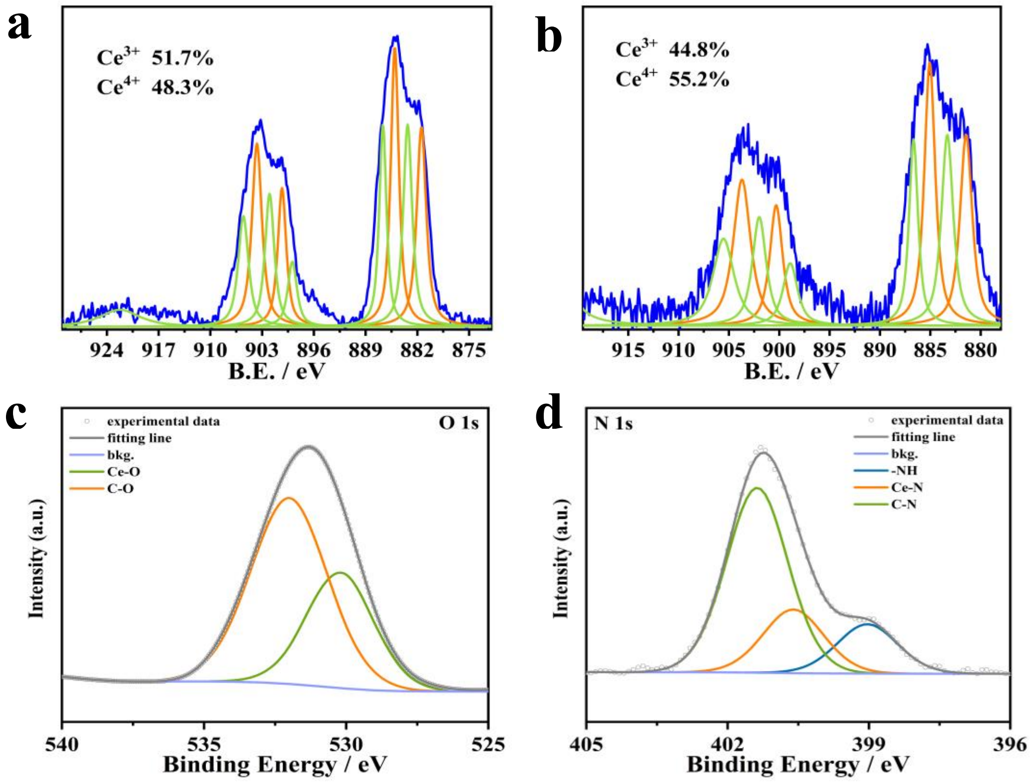

Supplementary Figure 17. XPS characterizes the valence distribution of CZ NCs a) and PCZ@PB NCs b). c) Characteristic map of oxygen elements. d) Characteristic map of nitrogen elements.

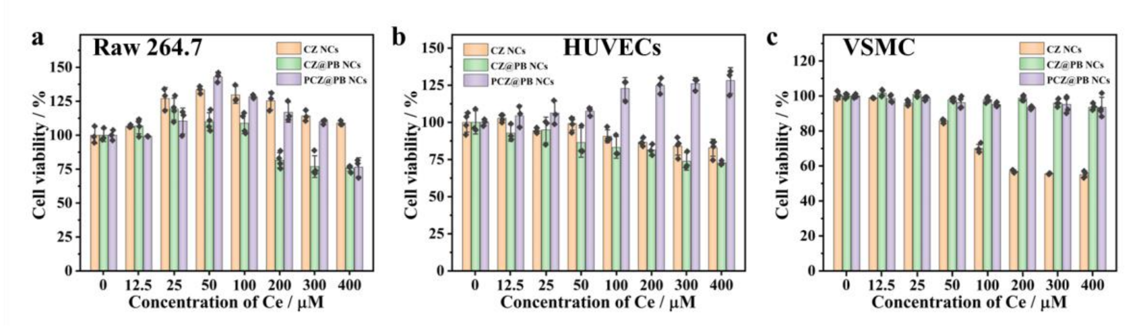

Supplementary Figure 18. Viability of a) RAW 264.7, b) HUVECs, c) VSMCs after 24 h treated with different concentrations of CZ NCs, CZ@PB NCs and PCZ@PB NCs. The experiments were repeated for three times (n = 3) and data were presented as mean ± s.d.

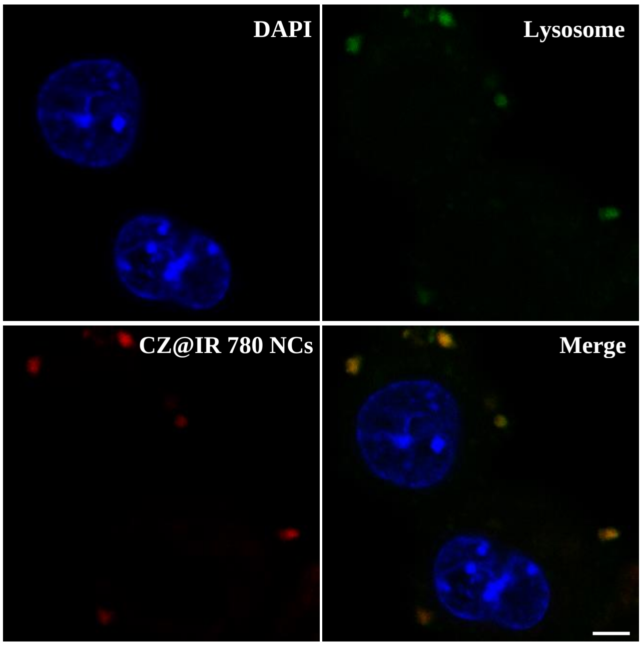

Supplementary Figure 19. Confocal fluorescent microscopy characterizes the internalization of CZ NCs by cells. The experiments were repeated for three times (n = 3) with similar results. Scale bar, 5 μm.

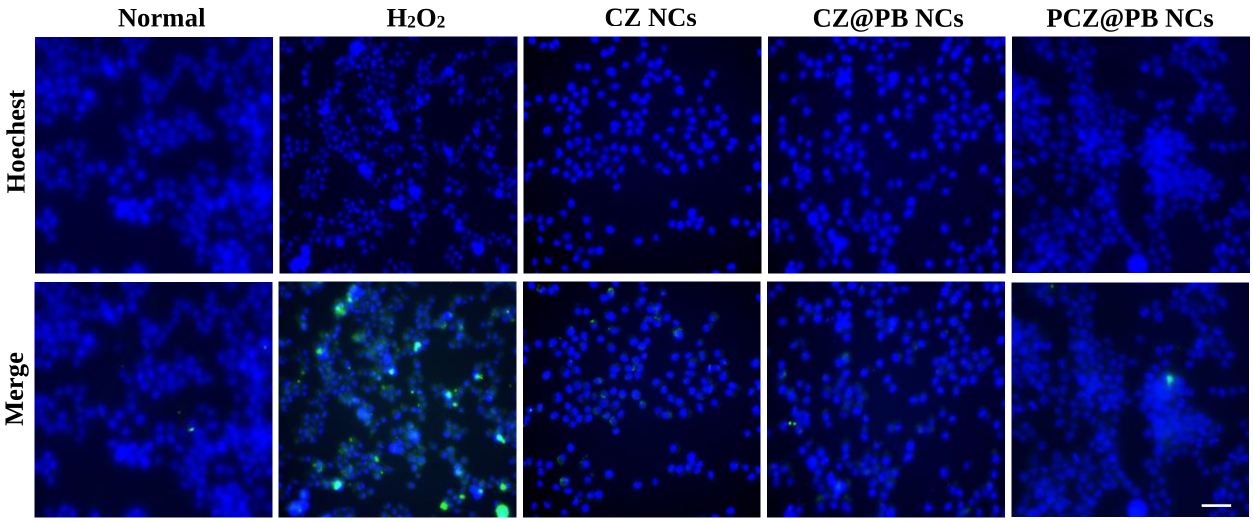

Supplementary Figure 20. Characterization of the ability of different formulations to eliminate H_2O_2 in cells (blue: hoechest, cell nucleus; green: DCFH-DA, H_2O_2). The experiments were repeated for three times (n = 3) with similar results. Scale bar, 200 μm.

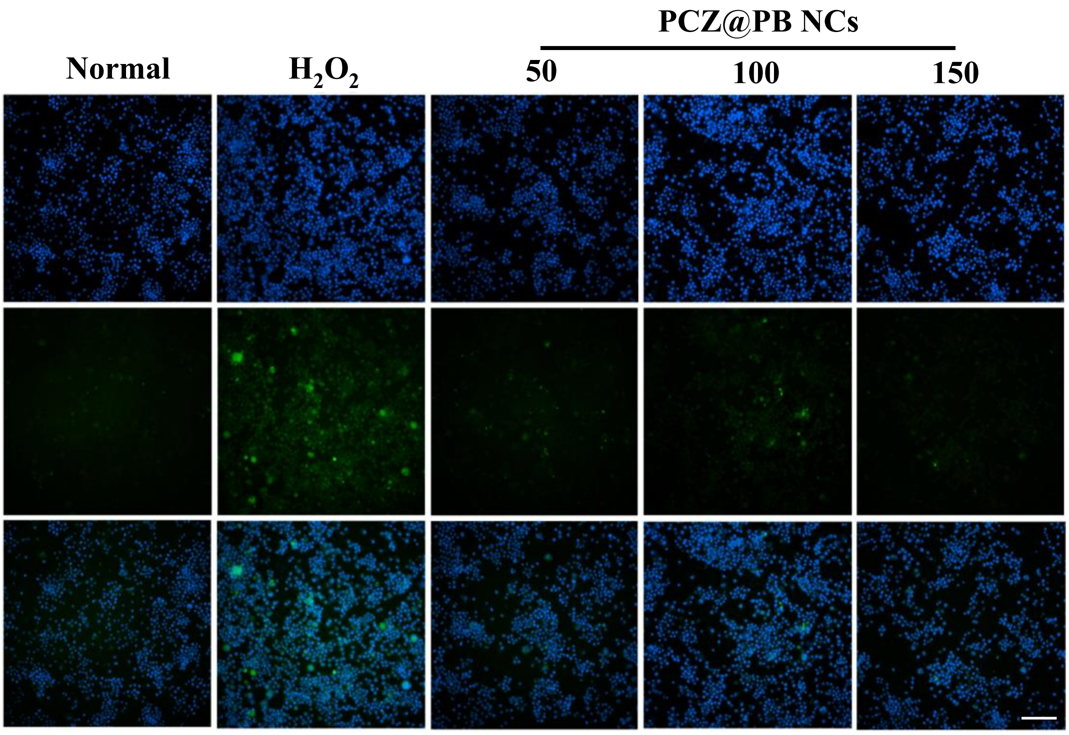

Supplementary Figure 21. Characterization of the ability of PCZ@PB NCs to eliminate H_2O_2 in cells (blue: DAPI, cell nucleus; green: DCFH-DA, H_2O_2). The experiments were repeated for three times (n = 3) with similar results. Scale bar, 200 μm.

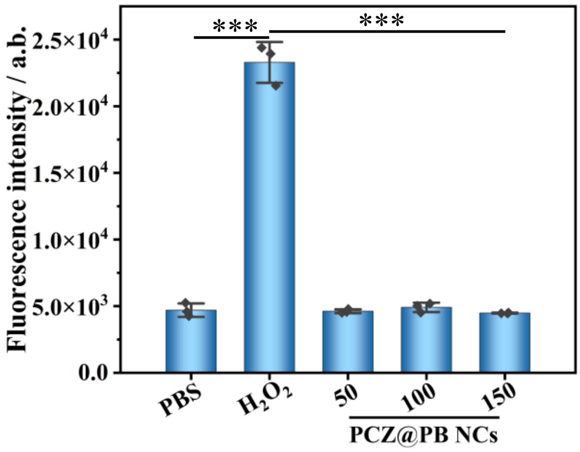

Supplementary Figure 22. Flow cytometry fluorescence intensity quantification of the ability of PCZ@PB NCs to eliminate H_2O_2 in cells. The experiments were repeated for three times (n = 3) and data were presented as mean ± s.d. Analysis of mean fluorescence intensity was performed using one-way ANOVA. *P ≤ 0.05, **P ≤ 0.01 and ***P ≤ 0.001. Specific P-values are shown in the source data. Source data are provided as source data files.

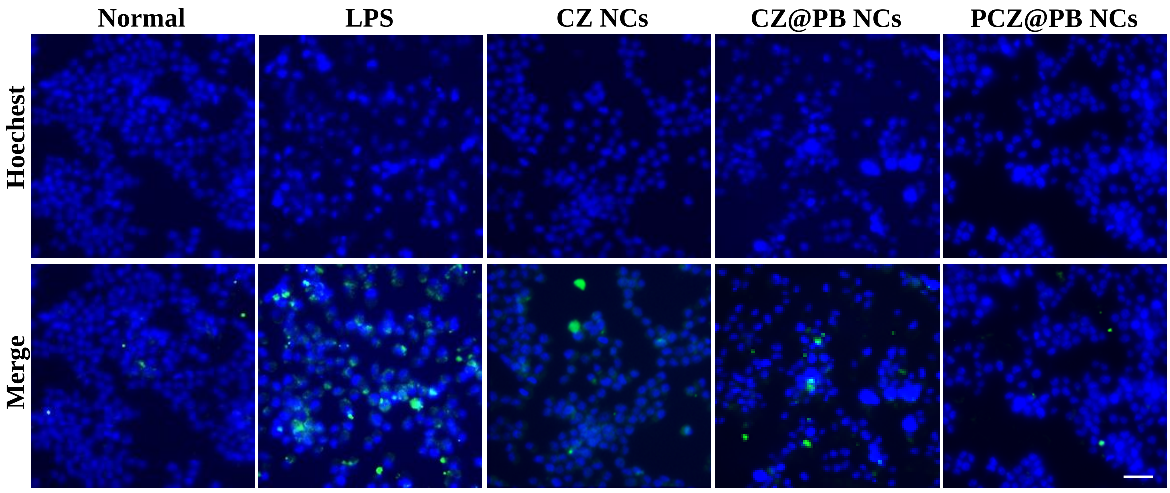

Supplementary Figure 23. Characterization of the ability of different formulations to resist LPS-stimulated intracellular ROS production (blue: hoechest, cell nucleus; green: DCFH-DA, ROS). The experiments were repeated for three times (n = 3) with similar results. Scale bar, 200 μm.

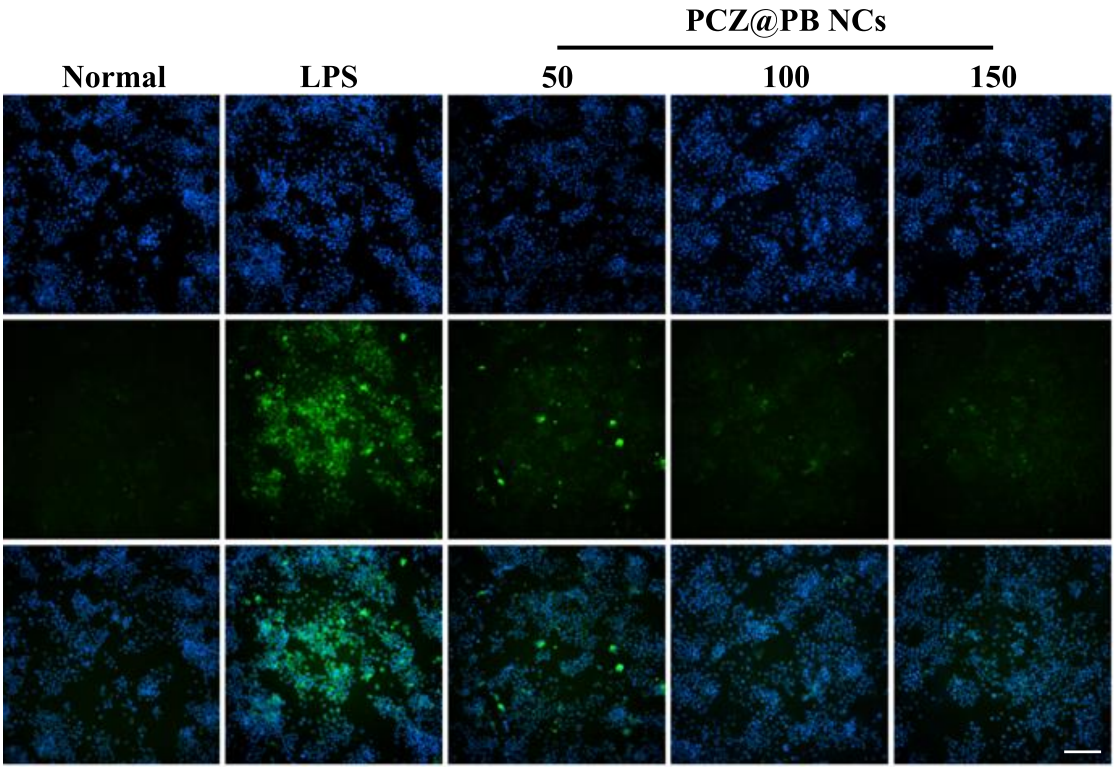

Supplementary Figure 24. Characterization of the ability of PCZ@PB NCs to resist LPS-stimulated intracellular ROS production (blue: DAPI, cell nucleus; green: DCFH-DA, ROS). The experiments were repeated for three times (n = 3) with similar results. Scale bar, 200 μm.

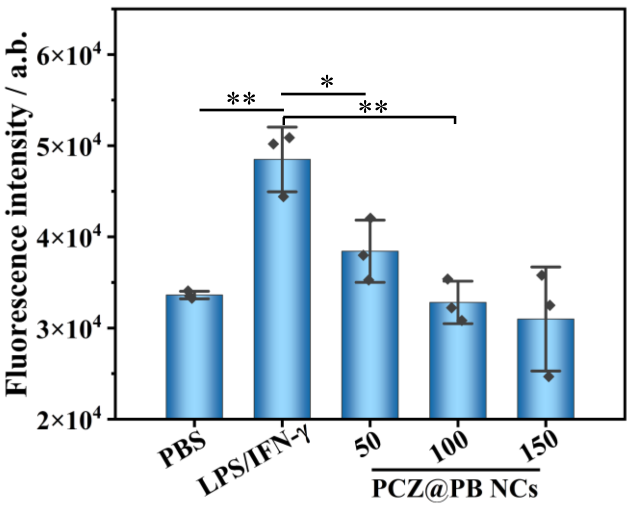

Supplementary Figure 25. Flow cytometry fluorescence intensity quantification of the ability of PCZ@PB NCs to e resist LPS-stimulated intracellular ROS production. The experiments were repeated for three times (n = 3) and data were presented as mean ± s.d. Analysis of mean fluorescence intensity was performed using one-way ANOVA. *P ≤ 0.05, **P ≤ 0.01 and ***P ≤ 0.001. Specific P-values are shown in the source data. Source data are provided as source data files.

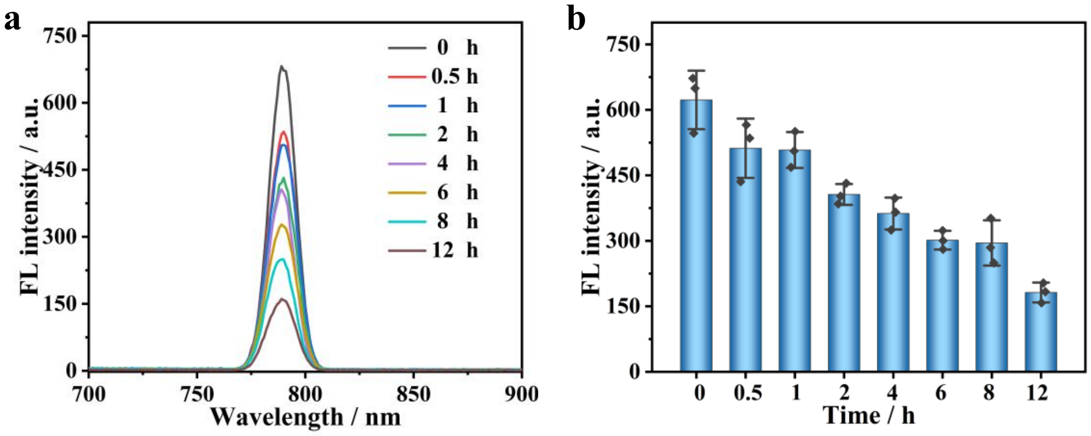

Supplementary Figure 26. *In vivo* pharmacokinetic performance of IR 780-labeled PCZ@PB NCs after i.v. injection in mice. (a) Fluorescence spectra of blood samples at each time point. (b) The quantified data of fluorescence intensities. The experiments were repeated for three times (n = 3) and data were presented as mean ± s.d.

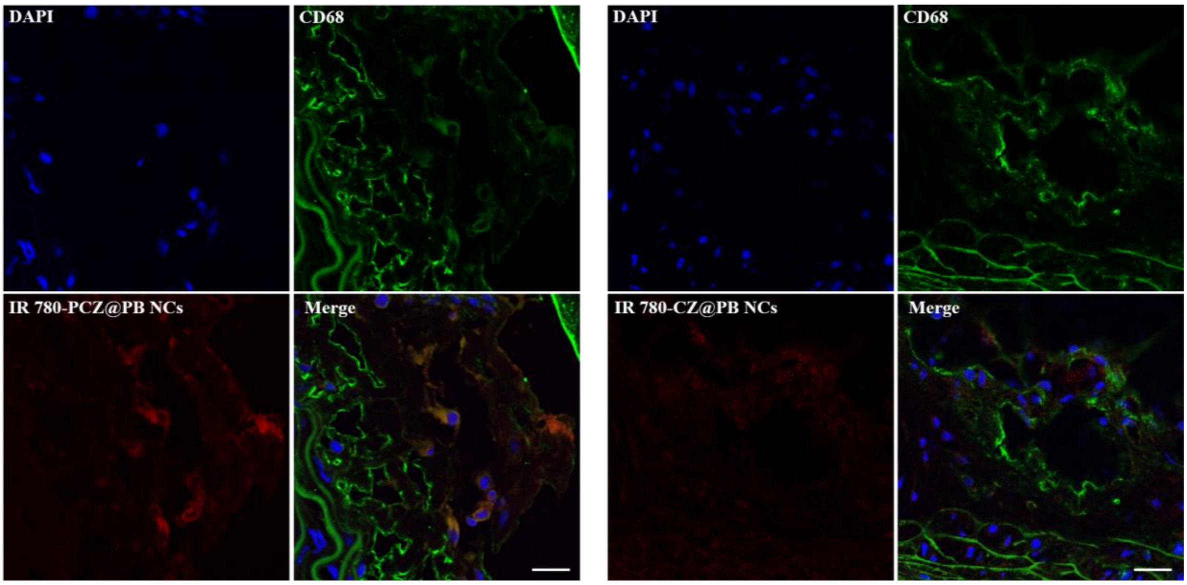

Supplementary Figure 27. Laser confocal characterization of PCZ@PB NCs colocalization with plaque macrophages. DAPI, nucleus; CD68, macrophage; PCZ@IR 780 NCs, PCZ@PB NCs. The experiments were repeated for three times (n = 3) with similar results. Scale bars, 20 μm.

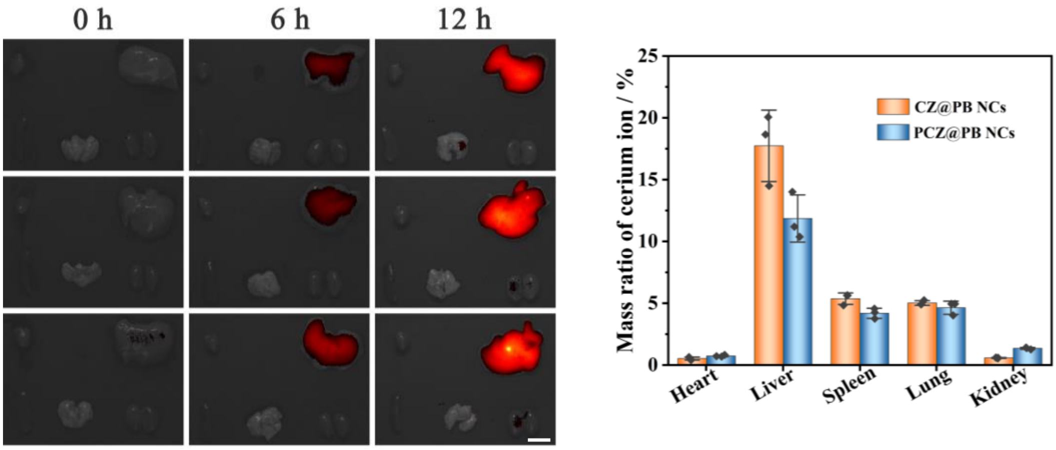

Supplementary Figure 28. On the left is the fluorescence distribution of IR 780 labeled PCZ@PB NCs in major organs in the body. On the right is the quantification of cerium ion concentration in major organs by ICP-MS. The experiments were repeated for three times (n = 3) and data were presented as mean ± s.d. Scale bar, 100 mm.

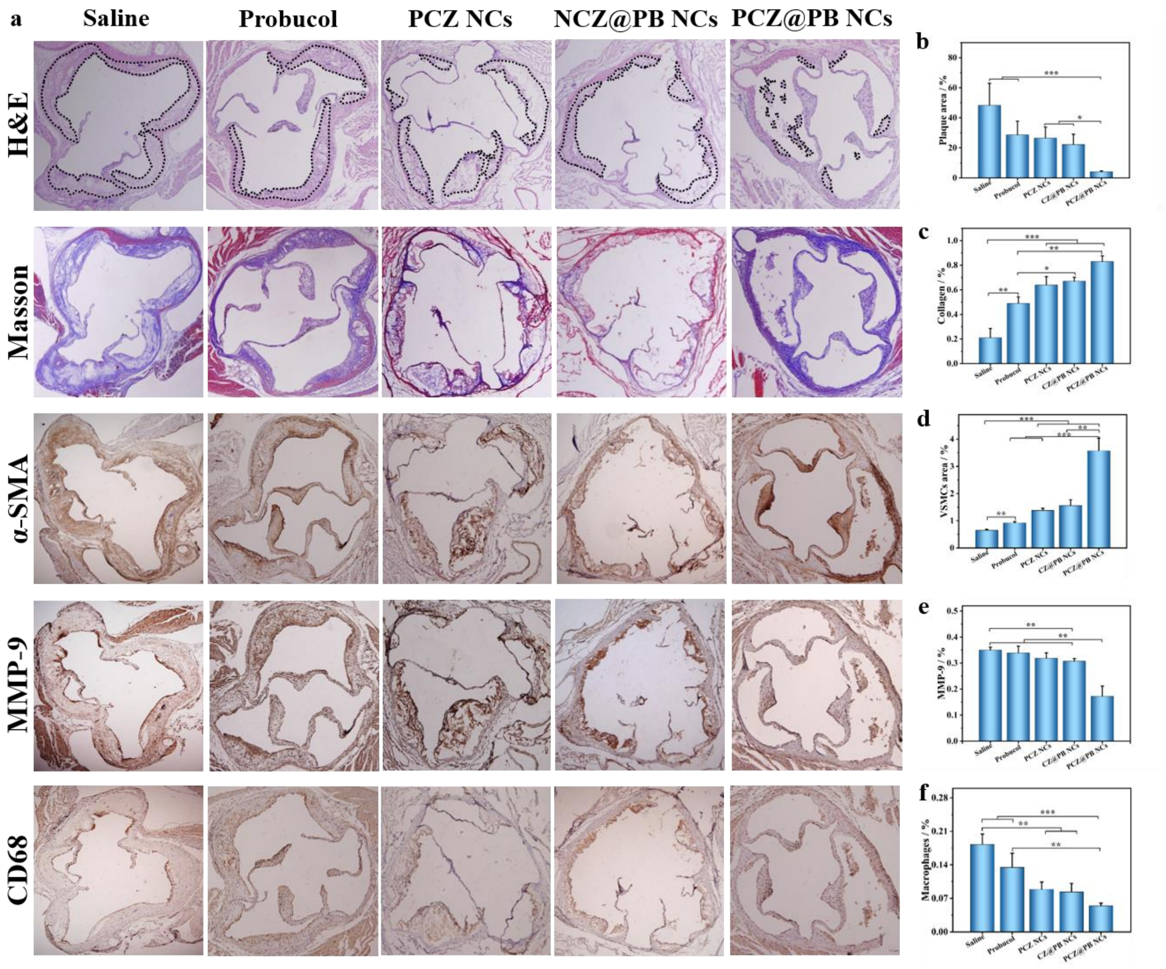

Supplementary Figure 29. Histochemistry analyses of aortic root sections from ApoE$^{-/-}$ mice after different treatments. (a) Representative images of aortic root sections stained with H&E, Masson's trichrome, antibody to α-SMA, antibody to CD68, and antibody to MMP-9. (b-f) Quantitative analysis of the plaque area (b), plaque collagen area (c), plaque VSMCs area (d), plaque MMP-9 area (e), and plaque macrophage area (f). The experiments were repeated for three times (n = 3) and data were presented as mean ± s.d. Statistical significance in b-f was calculated via one-way ANOVA. *P ≤ 0.05. Specific P-values are shown in the source data. Source data are provided as source data files.

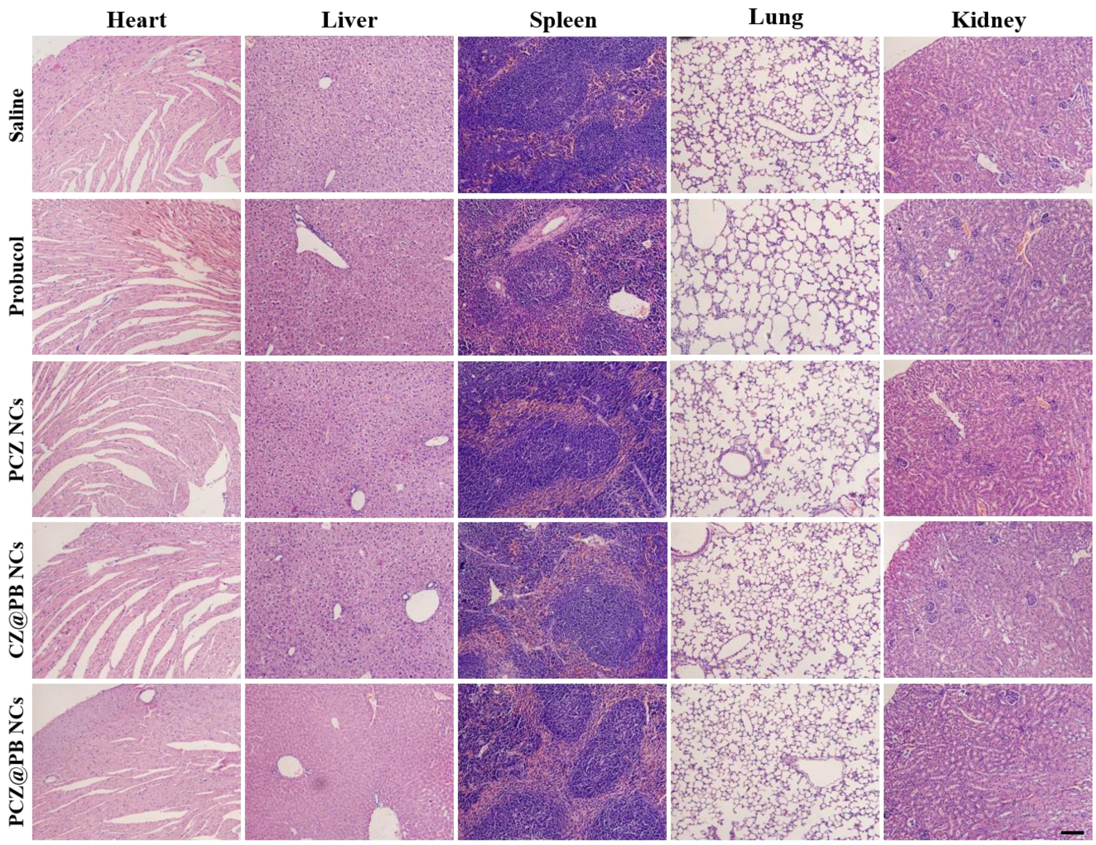

Supplementary Figure 30. H&E stained sections of major organs to assess *in vivo* safety. The experiments were repeated for three times (n = 3) with similar results. Scale bar, 100 μm.

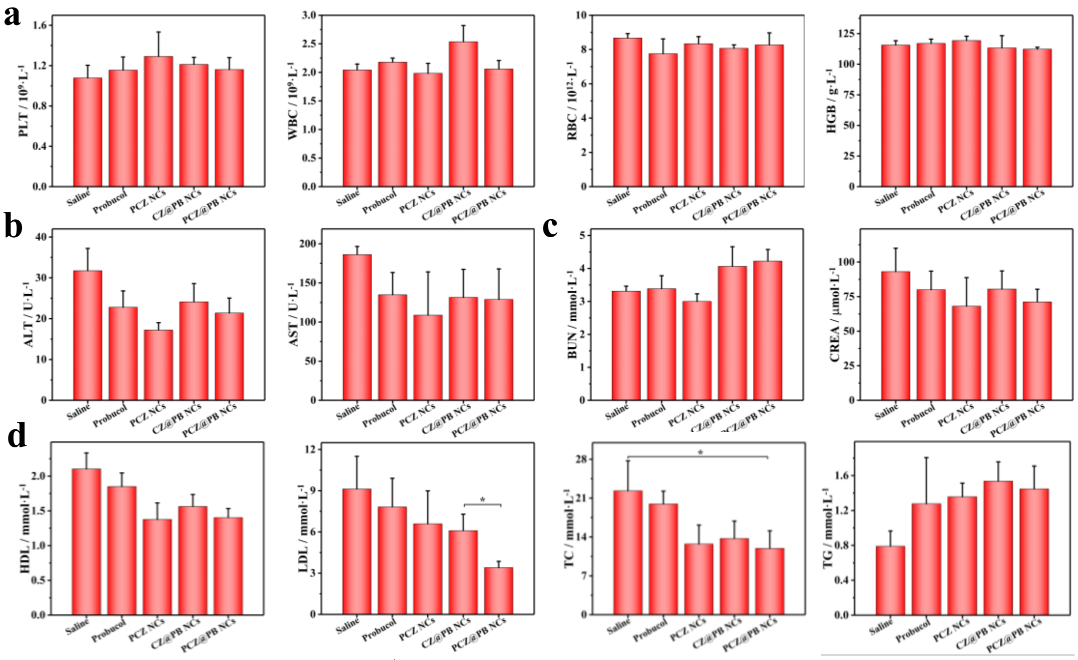

Supplementary Figure 31. Safety evaluations in ApoE$^{-/-}$ mice after long-term treatment with different formulations. a-b) Levels of typical hematological parameters (a) and biochemical markers relevant to hepatic (b) and kidney (c) functions in serum. d) Serum levels of typical lipid markers. PLT, platelet; WBC, white blood cell; RBC, red blood cell; HGB, hemoglobin; ALT, alanine aminotransferase; AST, aspartate aminotransferase; BUN, blood urea nitrogen; SCr, serum creatinine; HDL, high-density lipoprotein; LDL, low-density lipoprotein; TC, total cholesterol; TG, triglyceride. The experiments were repeated for three times (n = 3) and data were presented as mean ± s.d. Analysis of the concentrations of LDL and TC was performed using one-way ANOVA. *P ≤ 0.05. Specific P-values are shown in the source data. Source data are provided as source data files.

Supplementary Tables 1-4

Supplementary Table 1. List of reaction steps and product pictures of dual-ligand system.

Group name	①	②	③	④	⑤	⑥	⑦
**Step one**	**AL**	**AL**	**HMIM**	**HMIM**	**AL**	**H_2O (pH 9)**	**AL**
**Step two**	**$Ce(NO_3)_3$**	**HMIM**	**$Ce(NO_3)_3$**	**$Ce(NO_3)_3$**	**$Ce(NO_3)_3$**	**$Ce(NO_3)_3$**	**$Ce(NO_3)_3$**
**Step three**	**HMIM**	**$Ce(NO_3)_3$**	**AL**	**H_2O**	**H_2O**	**——**	**H_2O (pH 9)**
**Photos**	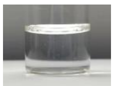	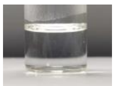	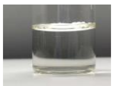	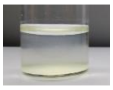	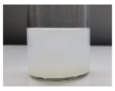	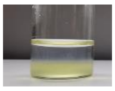	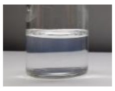

AL represents 1 mL of alendronic acid (10 mg/mL) and HMIM represents 1 mL of 2-methylimidazole (10 mg/mL). The concentration of $Ce(NO_3)_3$ was 0.1 M, and 100 μL was taken dropwise each time. H_2O was 1 mL of ultrapure water.

Supplementary Table 2. CZ NCs co-assembled with different drugs and analysis results of hydrated particle size of the products.

Name	Molecular weight	Average size	Nanoparticle formation
Chondroitin sulfate	479.37	523	√
Deferoxamine	656.79	798	√
Streptozocin	265.22	498	√
Orlistat	495.73	640	√
Rapamycin	914.17	700	√
Dopamine hydrochloride	153.18	299	√
Favipiravir	157.10	254	√
Mechlorethamine hydrochloride	192.51	376	√
5-Fluorouracil	130.08	295	√
Adriamycin	543.52	670	√
Tetracycline hydrochloride	480.90	373	√
Cyclophosphamide	279.10	406	√
Human serum albumin (HSA)	66 kD	376	√
Human hemoglobin (HGB)	226.23	397	√
Atorvastatin	558.64	20	√
Metformin hydrochloride	129.16	135	√
Indomethacin	357.70	269	√
Sulindac	356.41	523	√
Paclitaxel	853.91	3352	X

Supplementary Table 3. Results of qualitative and quantitative analysis of oxygen-containing functional groups.

O	Name	Peak BE	FWHM eV	Area (P) CPS.eV	Atomic %
	Ce-O	530.22	2.82	304111.75	35.2
	C-O	532.01	3.22	559110.92	64.8

Supplementary Table 4. Results of qualitative and quantitative analysis of nitrigen-containing functional groups.

N	Name	Peak BE	FWHM eV	Area (P) CPS.eV	Atomic %
	NH	399.03	1.52	4518.68	16.4
	Ce-N	400.61	1.52	5832.26	21.18
	C-N	401.37	1.54	17188.7	62.43

Supplementary Video 1-2

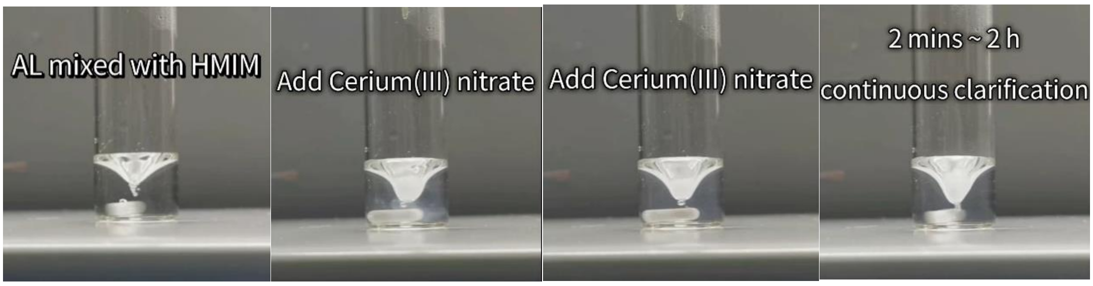

Supplementary Video 1. The preperation of dual-ligand ceria nanozyme platform (CHA). The CHA was obtained via the assembly of cerium ions, alendronate acid (AL) and HMIM.

Supplementary Video 2

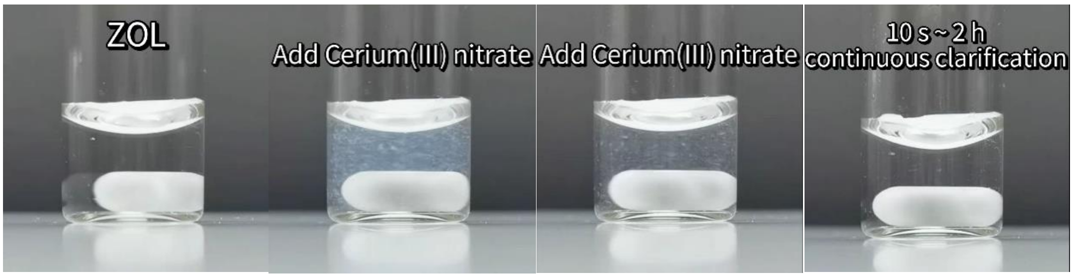

Supplementary Video 2. The preperation of single-ligand ceria-zoledronic acid nanocomposites (CZ NCs). The CZ NCs was obtained via the assembly of cerium ions and zoledronic acid.
