# Supplementary material for: Small molecule-assisted assembly of multifunctional ceria nanozymes for synergistic treatment of atherosclerosis
Source: Nat Commun. 2022 Nov 1;13:6528. doi: 10.1038/s41467-022-34248-y (PMC9626479; doi:10.1038/s41467-022-34248-y)
Supplement: Supplementary file 2 — Description of Additional Supplementary Files [file 41467_2022_34248_MOESM2_ESM.pdf]

Description of Additional Supplementary Files

File Name: Supplementary Video 1

Description: The preperation of dual-ligand ceria nanozyme platform (CHA). The CHA was obtained via the assembly of cerium ions, alendronate acid (AL) and HMIM.

File Name: Supplementary Video 2

Description: The preperation of single-ligand ceria-zoledronic acid nanocomposites (CZ NCs). The CZ NCs was obtained via the assembly of cerium ions and zoledronic acid.
