# Supplementary material for: Small molecule-assisted assembly of multifunctional ceria nanozymes for synergistic treatment of atherosclerosis
Source: Nat Commun. 2022 Nov 1;13:6528. doi: 10.1038/s41467-022-34248-y (PMC9626479; doi:10.1038/s41467-022-34248-y)
Supplement: Supplementary file 5 — Reporting Summary [file 41467_2022_34248_MOESM5_ESM.pdf]

nature portfolio

Corresponding author(s): Zhangyou Yang and Chao Yu

Last updated by author(s): Oct 9, 2022

Reporting Summary

Nature Portfolio wishes to improve the reproducibility of the work that we publish. This form provides structure for consistency and transparency in reporting. For further information on Nature Portfolio policies, see our Editorial Policies and the Editorial Policy Checklist.

Please do not complete any field with "not applicable" or n/a. Refer to the help text for what text to use if an item is not relevant to your study.
For final submission: please carefully check your responses for accuracy; you will not be able to make changes later.

Statistics

For all statistical analyses, confirm that the following items are present in the figure legend, table legend, main text, or Methods section.

n/a	Confirmed	
☐	☒	The exact sample size (*n*) for each experimental group/condition, given as a discrete number and unit of measurement
☐	☒	A statement on whether measurements were taken from distinct samples or whether the same sample was measured repeatedly
☐	☒	The statistical test(s) used AND whether they are one- or two-sided<br>*Only common tests should be described solely by name; describe more complex techniques in the Methods section.*
☒	☐	A description of all covariates tested
☐	☒	A description of any assumptions or corrections, such as tests of normality and adjustment for multiple comparisons
☐	☒	A full description of the statistical parameters including central tendency (e.g. means) or other basic estimates (e.g. regression coefficient) AND variation (e.g. standard deviation) or associated estimates of uncertainty (e.g. confidence intervals)
☐	☒	For null hypothesis testing, the test statistic (e.g. F, t, r) with confidence intervals, effect sizes, degrees of freedom and P value noted<br>*Give P values as exact values whenever suitable.*
☒	☐	For Bayesian analysis, information on the choice of priors and Markov chain Monte Carlo settings
☒	☐	For hierarchical and complex designs, identification of the appropriate level for tests and full reporting of outcomes
☒	☐	Estimates of effect sizes (e.g. Cohen's *d*, Pearson's *r*), indicating how they were calculated

Our web collection on statistics for biologists contains articles on many of the points above.

Software and code

Policy information about availability of computer code

Data collection	Data were collected with various laboratory instruments and equipments. Such as: Field emission scanning electron microscope (SEM) was measured on SU8020 (HITACHI, Japan). Inductively coupled plasma mass spectrometry (ICP-MS, Agilent 7500ce, America). Nikon Eclipse Ti-S inverted fluorescence microscope (Nikon Corporation, Japan). Upright microscope (Nikon ECLIPSE Ci, Nikon Corporation). Fully automatic chemiluminescence/fluorescence image analysis system (Tanon 5200, Shanghai Tanon). Portable dissolved oxygen analyzer (JPBJ-608, Shanghai INESA).
Data analysis	ImageJ_v1.8.0, Microsoft Office Home and Student 2019, ChemOfficeSuite2019, CytExpert Version 2.3.0.84, Prism 5, FlowJo_V10

For manuscripts utilizing custom algorithms or software that are central to the research but not yet described in published literature, software must be made available to editors and reviewers. We strongly encourage code deposition in a community repository (e.g. GitHub). See the Nature Portfolio guidelines for submitting code & software for further information.

Data

Policy information about availability of data

All manuscripts must include a data availability statement. This statement should provide the following information, where applicable:

- Accession codes, unique identifiers, or web links for publicly available datasets
- A description of any restrictions on data availability
- For clinical datasets or third party data, please ensure that the statement adheres to our policy

Provide your data availability statement here.

Field-specific reporting

Please select the one below that is the best fit for your research. If you are not sure, read the appropriate sections before making your selection.

☒ Life sciences ☐ Behavioural & social sciences ☐ Ecological, evolutionary & environmental sciences

For a reference copy of the document with all sections, see nature.com/documents/nr-reporting-summary-flat.pdf

Life sciences study design

All studies must disclose on these points even when the disclosure is negative.

Sample size	No statistical methods were used to pre-determine sample sizes. Based on the literature and our previous studies, for each experiment we used at least n = 3 replicates to calculate the statistical values for each analysis.
Data exclusions	No data were excluded from this study.
Replication	All experiments were repeated at least three times to reliably support the conclusions stated in the manuscript.
Randomization	The mice were randomly assigned to cages and then divided into individual experimental groups for further treatment.
Blinding	For the cell-based experiments, blinding was not performed because the investigator had to know the group to which the drug was administered or the group performing the assay. Blinding was not performed in the mouse experiments because the researchers needed to know the treatment group in order to administer the drug. However, none of these objective factors affect the conclusions of the article.

Reporting for specific materials, systems and methods

We require information from authors about some types of materials, experimental systems and methods used in many studies. Here, indicate whether each material, system or method listed is relevant to your study. If you are not sure if a list item applies to your research, read the appropriate section before selecting a response.

Materials & experimental systems

n/a	Involved in the study	
☐	☒	Antibodies
☐	☒	Eukaryotic cell lines
☒	☐	Palaeontology and archaeology
☐	☒	Animals and other organisms
☒	☐	Human research participants
☒	☐	Clinical data
☒	☐	Dual use research of concern

Methods

n/a	Involved in the study	
☒	☐	ChIP-seq
☐	☒	Flow cytometry
☒	☐	MRI-based neuroimaging

Antibodies

Antibodies used	NF-κB p65 antibody (catlog number: ab76302, clone number: EP2294Y, abcam);<br>TNF-α antibody (catlog number: ab183218, clone number: EPR19147, abcam);<br>MMP-9 antibody (catlog number: ab38898, abcam);<br>GAPDH antibody (catlog number: AF0006, beyotime)
Validation	All antibodies were commercially available and were validated by the supplier. All antibodies were used in the study according to the profile of manufacturers.

Eukaryotic cell lines

Policy information about cell lines

Cell line source(s)	The cell lines of human umbilical vein endothelial cell HUVEC and mouse macrophage cell line Raw 264.7 were purchased from Procell Life Science & Technology, China.
Authentication	Raw 264.7 was validated by the supplier by short tandem repeat (STR) analysis. HUVEC was identified by the supplier by CD31 immunofluorescence.
Mycoplasma contamination	All cell lines tested negative for mycoplasma contamination.

Commonly misidentified lines (See ICLAC register)	No commonly misidentified lines were used in this study.

Animals and other organisms

Policy information about studies involving animals; ARRIVE guidelines recommended for reporting animal research

Laboratory animals	Six-week-old male ApoE-/- mice were obtained from Cavens Laboratory Animals Co., China.<br>RRID: IMSR_JAX:00052<br>Mice were housed in an IVC system with a temperature of 23℃-25℃ and a humidity of 60%-70%. The light cycle of the animal room is 12 hours of light and 12 hours of darkness.
Wild animals	No wild animals were used.
Field-collected samples	This study did not involve samples collected from the field.
Ethics oversight	All animal studies were conducted in accordance with the regulations and guidelines of the Medical Research Ethics Committee of Chongqing Medical University. All animal experiments procedures and protocols were approved by the Experimental Animal Center of Chongqing Medical University (SCXK2018-0003).

Note that full information on the approval of the study protocol must also be provided in the manuscript.

Flow Cytometry

Plots

Confirm that:

- [ ] The axis labels state the marker and fluorochrome used (e.g. CD4-FITC).
- [x] The axis scales are clearly visible. Include numbers along axes only for bottom left plot of group (a 'group' is an analysis of identical markers).
- [x] All plots are contour plots with outliers or pseudocolor plots.
- [x] A numerical value for number of cells or percentage (with statistics) is provided.

Methodology

Sample preparation	The cells were rinsed three times with PBS and then detached from the well plate for flow cytometry analysis.
Instrument	CytoFLEX flow cytometer
Software	CytExpert Version 2.3.0.84 was used for flow cytometry data collection, FlowJo_V10 was used for flow cytometry data analysis.
Cell population abundance	Flow cytometry was used for quantitative analysis only. At least 10,000 cells were analyzed for each sample.
Gating strategy	Generally, the initial cell population was gated by FSC/SSC. The gate was set to exclude cell debris and dead cells.

- [ ] Tick this box to confirm that a figure exemplifying the gating strategy is provided in the Supplementary Information.

This checklist template is licensed under a Creative Commons Attribution 4.0 International License, which permits use, sharing, adaptation, distribution and reproduction in any medium or format, as long as you give appropriate credit to the original author(s) and the source, provide a link to the Creative Commons license, and indicate if changes were made. The images or other third party material in this article are included in the article's Creative Commons license, unless indicated otherwise in a credit line to the material. If material is not included in the article's Creative Commons license and your intended use is not permitted by statutory regulation or exceeds the permitted use, you will need to obtain permission directly from the copyright holder. To view a copy of this license, visit http://creativecommons.org/licenses/by/4.0/
